# Level set-based image segmentation of $\mu$CT scanned oak micro-structures with an analysis of morphological features

**DOI:** 10.1007/s00226-025-01660-8

**Published:** 2025-05-28

**Authors:** M. A. Livani, A. S. J. Suiker, E. Bosco

**Affiliations:** Department of the Built Environment, Chair of Applied Mechanics, Eindhoven University of Technology, P.O. Box 513, 5600 MB Eindhoven, The Netherlands

## Abstract

A three-dimensional level set-based image segmentation method is presented for a robust identification and accurate characterization of the different cell types defining complex wood micro-structures. The method can be applied to arbitrary wood species, and in this contribution is elaborated for oak. The evolution of the level set function and the corresponding boundary conditions are rigorously derived from a variational framework based on the Local Chan-Vese energy functional. The application of the level-set image segmentation approach enables to distinguish the cell wall material from the cell cavities. The cell material objects are subsequently segmented into axial cell objects and ray parenchyma cell objects that are oriented in the longitudinal and radial material directions of oak wood, respectively. This additional segmentation step facilitates the collection of statistical information on the inner cell dimensions and wall thickness of axial cells and ray parenchyma cells from images taken across principal material planes of the oak micro-structure. The performance and results of the image segmentation method are analyzed by using as input detailed micro-structural images of two representative oak samples containing a single growth ring, as obtained from X-ray micro-computed tomography experiments. The assessment of the robustness and convergence behaviour of the image segmentation method shows that the method converges very fast into a unique oak micro-structure that is independent of the initial configuration selected. The accuracy of the image segmentation result is shown through a comparison with the results obtained by two other image segmentation methods presented in the literature, and by visualizing and identifying small-scale morphological features within oak growth rings in great detail. The computational cost of the image segmentation method is evaluated by comparing its performance on CPU and GPU hardware. Additionally, a statistical analysis is carried out of the maximum and minimum inner cell diameters and the cell wall thickness of the various axial cells—fibers and axial parenchyma, earlywood vessels, latewood vessels—and ray parenchyma cells defining the micro-structure of the oak growth ring samples. The density histograms constructed for these geometrical parameters provide their statistical spread and most frequent value, which are quite similar for the two oak samples and are in good agreement with other experimental data reported in the literature. The oak micro-structures identified and characterized by the present image segmentation method may serve as input for dedicated finite element models that compute their mechanical/physical behaviour as a function of the geometrical and physical properties of the individual cells.

## Introduction

Wood is a complex, heterogeneous material composed of diverse cell types with distinct geometrical and physical properties (Ross 2010; Rowell 2005). The hierarchical material structure of wood involves length scales ranging from the macroscopic, structural scale down to the nanoscopic, molecular scale, where the morphological features at lower scales determine the effective mechanical and physical properties of wood at the macro-scale (Borrega et al. 2015; Malek and Gibson 2015; Livani et al. 2021, 2023, 2025). The biological nature of wood, however, provides a variability to these morphological features, which are species-dependent and also different for softwoods and hardwoods. Specifically, softwoods are characterized by an approximately regular cell structure of longitudinal tracheids and ray parenchyma cells (Richter et al. 2004; Scheperboer et al. 2019), while hardwoods are defined by rather complex cell structures of fibers, axial parenchyma, ray parenchyma, earlywood vessels and latewood vessels (Wheeler et al. 1989; Kim and Daniel 2016; Livani et al. 2023).

A detailed characterization of the geometrical properties of the different cell types in wood requires the application of advanced imaging and image processing techniques (Perré 2005; Paris et al. 2015; Perré et al. 2016; Chakkour and Perré 2024). Typical imaging techniques are: light microscopy, electron microscopy, X-ray micro-computed tomography ($\mu$CT) imaging and nuclear magnetic resonance imaging (Murphy and Davidson 2012; Daniel 2016; Bastani et al. 2016; Luimes et al. 2018; Livani et al. 2023). Of these techniques, $\mu$CT scanning is used for visualizing interior features of wood micro-structures and for obtaining digital information on their 3D micro-structural geometries and properties, where samples can be imaged with voxel sizes as small as one tenth of a micrometer, and objects can be scanned up to 200 mms in diameter (Peng et al. 2015; Brereton et al. 2015; Koddenberg and Militz 2018). The greyscale intensity values of the voxels denote the local material density, which can be used as input for an image segmentation method to identify the individual micro-structural phases composing the material.

Image segmentation is the process of converting an image into a collection of distinct objects/phases (i.e., sets of pixels or voxels), where each object is assigned a label that refers to its specific properties (Tan 2016). In the literature, various image segmentation methods have been developed, such as level set-based image segmentation (Ramlau and Ring 2007), watershed segmentation (Salman 2006; Lin et al. 2006), grey level thresholding (Otsu 1979), spline-based segmentation (Chen et al. 2009; Mayo et al. 2010) and image segmentation methods based on neural networks (Lorenzoni et al. 2020; Nefs et al. 2023). Level sets are especially suitable for being applied in image segmentation, as they provide accurate descriptions for configurations characterized by phase boundaries with complex shapes (with sharp corners and cusps), and generally behave in a numerically robust fashion, with the converged segmentation result being relatively insensitive to image noise and the initial configuration assumed (Lin et al. 2004). The process of level set-based image segmentation starts with the level set function defining the boundary between the phases of the initial two-phase configuration selected. The level set function subsequently evolves, based on local changes in the image intensity, until it represents a steady phase boundary that corresponds with the desired image object boundary. The process is guided by a level set evolution equation, which can be derived from the principle of minimization of an “energy” functional. The energy functional is constructed from image information (i.e., global and local spatially-averaged voxel intensities), and is designed such that it achieves a balance between the smoothness of the boundary and its adherence to the image intensity (Chan and Vese 1999, 2001; Wang et al. 2010).

Utilizing the above-mentioned benefits of level sets, in the present work a three-dimensional level set-based image segmentation method is presented that can be used for a robust identification and accurate characterization of the diverse cell types defining complex (oak) wood micro-structures. The evolution of the level set function and the corresponding boundary conditions are rigorously derived from a variational framework based on the Local Chan-Vese energy functional presented in Wang et al. (2010). The application of the level-set image segmentation approach enables to distinguish the cell (wall) material from the cell cavities. Through the addition of a skeleton-based segmentation step, the cell material objects are subsequently segmented into axial cell objects and ray parenchyma cell objects that are oriented in the longitudinal and radial material directions of oak wood, respectively. This additional segmentation step facilitates the collection of statistical information on the inner cell dimensions and wall thickness of axial cells and ray parenchyma cells from images taken across the principal material planes of the oak micro-structure. The performance and results of the image segmentation method are analyzed by using as input detailed micro-structural images of two representative oak samples containing a single growth ring, as obtained from X-ray micro-computed tomography experiments. The robustness and convergence behaviour of the image segmentation method are investigated, and the accuracy is demonstrated. For this purpose, the results of the image segmentation method are compared to those obtained by two other image segmentation methods presented in the literature, and the difference in computational cost of the image segmentation method is assessed on central processing unit (CPU) and graphics processing unit (GPU) hardware. Additionally, the practical applicability of the method for the wood science community is demonstrated by performing a statistical analysis of the maximum and minimum inner cell diameters and the cell wall thickness of the various axial cells—fibers and axial parenchyma, earlywood vessels, latewood vessels—and ray parenchyma cells defining the micro-structures of the oak growth ring samples.

In summary, the aim and novelty of the present work thus comprise the following three main aspects: (i) a rigorous mathematical derivation of the governing equations required for a robust implementation of a three-dimensional level-set image segmentation method based on the Local Chan-Vese energy potential, (ii) the augmentation of the method with a skeleton-based image segmentation step to uniquely identify and statistically characterize the various cell geometries defining the complex oak wood micro-structures, (iii) a demonstration of the accuracy, robustness, convergence behaviour, efficiency and applicability of the image segmentation method using detailed oak micro-structures obtained from $\mu$CT imaging, and including comparison studies with regard to other image segmentation methods and the use of CPU and GPU hardware. The complex, three-dimensional oak micro-structures identified and characterized with the present method can serve as input for dedicated finite element models that simulate in detail the local interactions at the micro-structural level, and from which the effective constitutive properties of oak can be determined as a function of the geometrical and physical microstructural features by applying numerical homogenization techniques, see Livani et al. (2023, 2025) for more details.

The paper is organized as follows. Sect. ""Level set-based image segmentation method"" presents the variational framework based on the Local Chan-Vese energy functional, which results in the evolution equation of the level set function and the corresponding boundary conditions. Subsequently, the additional, skeleton-based segmentation step into axial cells and ray parenchyma cells is formulated, followed by a description of the determination of the inner cell wall dimensions and cell wall thickness of these cell types. Section "Performance and results of image segmentation method" treats the X-ray micro-computed tomography experiments applied for obtaining detailed micro-structural images of two growth ring samples. These images are next used as input for the level set-based image segmentation method to assess its robustness and convergence behaviour. Additionally, the accuracy of the image segmentation result is shown through a comparison with the results obtained by two other image segmentation methods presented in the literature, and by visualizing and identifying small-scale morphological features within oak growth rings in great detail. The computational cost of the image segmentation method is evaluated by comparing its performance on CPU and GPU hardware. Finally, a statistical analysis is carried out of the maximum and minimum inner cell diameters and the cell wall thickness of the various cell types present in the micro-structural samples. Section "Conclusions" summarizes the conclusions of the work.

## Level set-based image segmentation method

In this section, a three-dimensional level set-based image segmentation method is presented that can be applied to the identification and geometrical characterization of the different cell types defining (oak) wood micro-structures. Section "Level set function" reviews the main characteristics of a level set function within the context of image segmentation. Section "Local Chan-Vese energy functional" presents the basic ingredients of the image segmentation method, in accordance with the “energy” functional originally proposed by Chan and Vese (Chan and Vese 1999, 2001) and subsequently enhanced in Wang et al. (2010). In addition to global image information, the enhanced energy functional considers local image information, which allows to adequately segment images with significant intensity inhomogeneity. In Sect. "Evolution of level set function and boundary conditions", the evolution equation of the level set function and the corresponding boundary conditions of the scanned domain are rigorously derived from a variational framework that minimizes the energy functional. An incremental update algorithm is formulated, which is based on combining an explicit time integration scheme for the time-discretized evolution of the level set function with a finite difference scheme for the discretization of its spatial derivatives. In Sect. "Skeleton-based image segmentation into axial cells and ray parenchyma cells" the level set image segmentation method is extended with an additional step in which the segmented cells are separated into axial cells and ray parenchyma cells oriented in the longitudinal and radial material directions of oak wood, respectively. The application of this additional segmentation step enables collecting statistical information on the inner cell dimensions and cell wall thicknesses of various cell types, as explained in Sect. "Statistical information on geometrical micro-structural features".

### Level set function

**Fig. 1:**
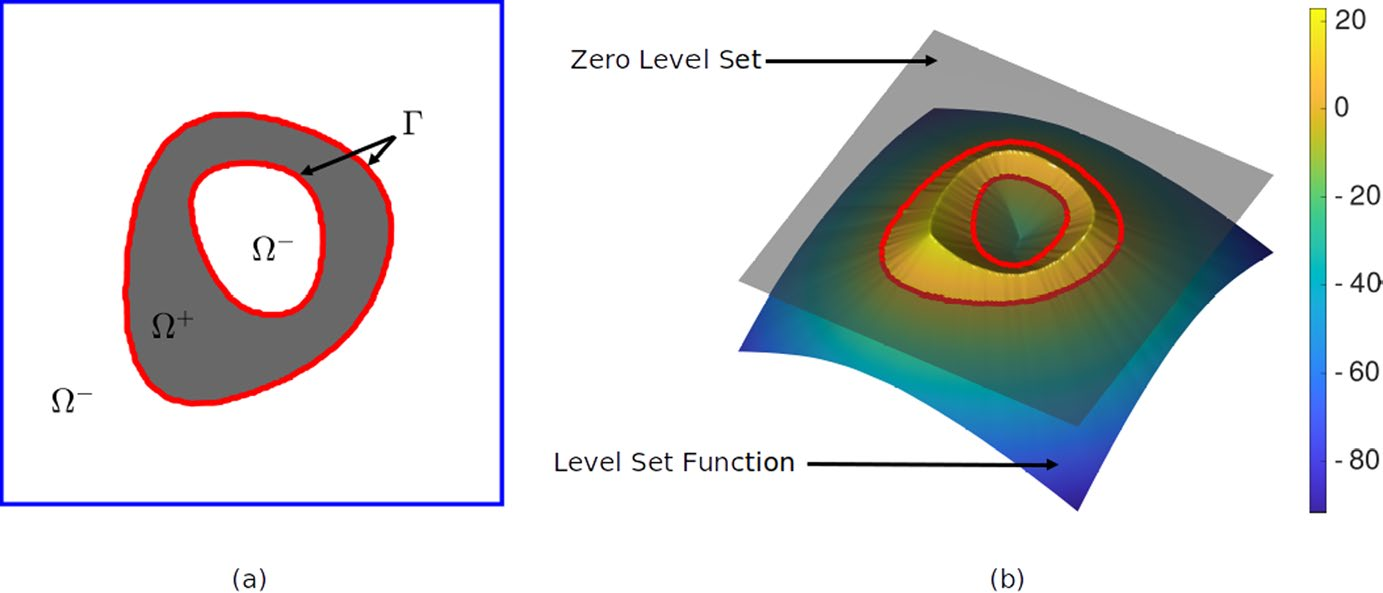
Schematic representation of level set-based phase segmentation. **a** Domain $\Omega$ composed of two subdomains $\Omega ^+$ (grey colour) and $\Omega ^-$ (white colour) with distinct phases, which are separated by a phase boundary $\Gamma$ (red solid line). **b** Contour plot of the level set function $\phi (\textbf{x})$, with its zero value defining the phase boundary $\Gamma$ indicated by a red solid line. The positive and negative values of $\phi (\textbf{x})$ (represented here in dimensionless units) are reflected by the colours yellow and green/blue, respectively (colour figure online)

Consider a domain $\Omega$ composed of two subdomains $\Omega ^+$ and $\Omega ^-$ with distinct pixel (2D) or voxel (3D) intensities, which are referred to as the *phases*, see Fig. 1a. The objective is to identify the geometries of these phases by applying a segmentation method based on an evolving level set function $\phi : \Omega \longrightarrow \mathbb {R}$ (Sethian 1999; Henri et al. 2022). In order to initialize the level set function, the Euclidean distance is computed from each pixel/voxel location $\textbf{x}$ to the *closest point* on the phase boundary $\Gamma$ separating the two phases. This leads to the function $\text {dist}(\textbf{x}, \Gamma )$, which allows to express the level set function as a signed distance function by assigning positive and negative signs to $\text {dist}(\textbf{x}, \Gamma )$ when $\textbf{x}$ is, respectively, located in the subdomains $\Omega ^+$ and $\Omega ^-$, i.e.,

1 $$\begin{aligned} \phi (\textbf{x}) = {\left\{ \begin{array}{ll} +\text {dist}(\textbf{x}, \Gamma ) & \text {for } \textbf{x} \in \Omega ^+ \, ,\\ -\text {dist}(\textbf{x}, \Gamma ) & \text {for } \textbf{x} \in \Omega ^- \, . \\ \end{array}\right. } \end{aligned}$$

Accordingly, the location of the phase boundary $\Gamma$ between the subdomains $\Omega ^+$ and $\Omega ^-$ follows from

2 $$\begin{aligned} \Gamma = \{ \textbf{x} \mid \phi (\textbf{x}) = 0 \}. \end{aligned}$$

The level set function $\phi (\textbf{x})$ and its zero value defining the phase boundary $\Gamma$ are displayed in Fig. 1b by a surface contour and a red solid line, respectively, where the positive and negative values of $\phi$ (represented here in dimensionless units) are reflected by the respective colours yellow and green/blue.

### Local Chan-Vese energy functional

The level set function $\phi$ given by Eq. (1) evolves due to local changes in the image intensity, until the phase boundary $\Gamma$ defined by Eq. (2) becomes stationary by reaching the desired image object boundary. The evolution of the level set function $\phi$ is described by an Euler-Lagrange equation, which follows from defining an appropriate “energy” functional and minimizing this functional with respect to $\phi$. In this work, the *Local Chan-Vese* energy functional is adopted, which is an extension of the original Chan-Vese energy functional (Chan and Vese 1999, 2001) by incorporating, in addition to *global* image information, *local* image information to adequately segment images with significant *intensity inhomogeneity* (Wang et al. 2010). Accordingly, the energy functional has the form (Wang et al. 2010)

3 $$\begin{aligned} E(c_1,c_2,d_1,d_2,\phi , \varvec{\nabla } \phi ) \, = \, \alpha E^G(c_1,c_2, \phi ) \, + \, \beta E^L(d_1,d_2, \phi ) \, + \, E^R(\phi , \varvec{\nabla } \phi ) \, , \end{aligned}$$

in which $E^G$ and $E^L$ respectively are the global and local energy terms, and $E^R$ is a regularization term that warrants that the level set function, Eq.(1), during its evolution remains i) smooth and ii) close to a signed distance function. These properties help to minimize the necessity for a reinitialization of the image segmentation process. Further, $\varvec{\nabla } \phi$ is the gradient of the level set function, and the parameters $\alpha$ and $\beta$ quantify the contributions of the global and local energy terms. The values of these parameters are commonly selected in accordance with the level of intensity inhomogeneity present in the image. The parameters $c_1$ and $c_2$ represent the averages of the (inhomogeneous) image intensities in the subdomains $\Omega ^+$ and $\Omega ^-$ shown in Fig. 1, and $d_1$ and $d_2$ are the averages of image intensity measures (to be specified below) across *local regions* of $\Omega ^+$ and $\Omega ^-$ in which the intensity is approximately homogeneous. The global energy term $E^G$ appearing in Eq. (3) is specified as (Wang et al. 2010)

4 $$\begin{aligned} E^G(c_1,c_2,\phi ) =\int _{\Omega } \left( I(\textbf{x})-c_1 \right) ^2 H_{\epsilon } \left( \phi (\textbf{x}) \right) \, \textrm{d}\Omega \, + \, \int _{\Omega } \left( I(\textbf{x})-c_2 \right) ^2 \left( 1- H_{\epsilon } \left( \phi (\textbf{x}) \right) \right) \, \textrm{d}\Omega \, , \end{aligned}$$

where $I(\textbf{x})$ is the image intensity at location $\textbf{x}$. Further, the average image intensity values $c_1$ and $c_2$ in the respective subdomains $\Omega ^+$ and $\Omega ^-$ are computed as

5 $$\begin{aligned} \begin{array}{lclcl} \vspace*{2mm} c_1 & = & \displaystyle {\frac{\int _{\Omega } I(\textbf{x}) H_{\epsilon } \left( \phi (\textbf{x}) \right) \textrm{d}\Omega }{\int _{\Omega } H_{\epsilon } \left( \phi ( \textbf{x}) \right) \textrm{d}\Omega } \, , } \\ c_2 & = & \displaystyle { \frac{\int _{\Omega } I(\textbf{x}) \left( 1 - H_{\epsilon } \left( \phi (\textbf{x}) \right) \right) \textrm{d}\Omega }{\int _{\Omega } \left( 1- H_{\epsilon } \left( \phi (\textbf{x}) \right) \right) \textrm{d}\Omega } \, , } \end{array} \end{aligned}$$

where the regularized Heaviside function $H_{\epsilon }(\phi )$ is given by (Wang et al. 2021; Yu et al. 2013)

6 $$\begin{aligned} H_{\epsilon }(\phi ) = \frac{1}{2}+\frac{1}{\pi }\textrm{arctan} \! \left( \frac{\phi }{\epsilon } \right) \, , \end{aligned}$$

with $\epsilon$ a parameter that regularizes the Heaviside function near $\phi =0$. In addition, the local energy term appearing in Eq. (3) is expressed by (Wang et al. 2010)

7 $$\begin{aligned} \vspace*{1mm} E^L(d_1,d_2,\phi )= & \int _{\Omega } \left( g_k * I(\textbf{x}) - I(\textbf{x}) - d_1 \right) ^2 H_{\epsilon }\left( \phi (\textbf{x}) \right) \textrm{d}\Omega \nonumber \\ + & \int _{\Omega } \left( g_k * I(\textbf{x}) - I(\textbf{x}) - d_2 \right) ^2 \left( 1 - H_{\epsilon } \left( \phi (\textbf{x}) \right) \right) \textrm{d}\Omega \,. \end{aligned}$$

Here, the averaging convolution operation $g_k * I(\textbf{x})$ determines the mean intensity within a *local*, cuboidal region centred around a cuboidal voxel with geometrical centre $\textbf{x}$, in accordance with (Schumacher 1991; Sun et al. 2016)

8 $$\begin{aligned} g_k * I(\textbf{x}) = \frac{1}{(2n+1)^3} \sum _{i=-n}^{n} \sum _{j=-n}^{n} \sum _{k=-n}^{n} I(\textbf{x} + i\textbf{e}_1 + j \textbf{e}_2 + k \textbf{e}_3 ) \, , \end{aligned}$$

which smoothens the image and reduces the impact of strong, local intensity variations. In Eq. (8), $\textbf{e}_1$, $\textbf{e}_2$ and $\textbf{e}_3$ are the orthonormal base vectors of a local Cartesian coordinate system within the cuboidal region. By scaling the edge length *h* of the cuboidal voxels to unity, and placing the origin of the local Cartesian coordinate system at the voxel centre $\textbf{x}$, the integer values *i*, *j* and *k* uniquely denote the individual voxels in the three spatial directions. Note that the intensity within an individual voxel is uniform and that the kernel size (i.e., the size of the cuboidal region) equals $(2n+1)\times (2n+1)\times (2n+1)$ voxels. The kernel size has to be properly selected to cover sufficient object and background voxels, as a result of which the sensitivity to noise remains limited (Wang et al. 2010). Additionally, the parameters $d_1$ and $d_2$ in Eq. (7) are the averages of the differences between the convolved image intensities and the original image intensities in the respective subdomains $\Omega ^+$ and $\Omega ^-$:

9 $$\begin{aligned} \begin{array}{lcl} \vspace*{2mm} d_1 & = & \displaystyle { \frac{\int _{\Omega } \left( g_k * I(\textbf{x}) - I(\textbf{x}) \right) H_{\epsilon } \left( \phi (\textbf{x}) \right) \textrm{d}\Omega }{\int _{\Omega } H_{\epsilon } \left( \phi (\textbf{x}) \right) \textrm{d}\Omega } } \, , \\ d_2 & = & \displaystyle { \frac{\int _{\Omega } \left( g_k * I(\textbf{x}) - I(\textbf{x}) \right) \left( 1 - H_{\epsilon } \left( \phi \left( \textbf{x} \right) \right) \right) \textrm{d}\Omega }{\int _{\Omega } \left( 1- H_{\epsilon } \left( \phi (\textbf{x}) \right) \right) \textrm{d}\Omega } } \, . \end{array} \end{aligned}$$

Further, the regularization term $E^R$ appearing in Eq. (3) has the form (Wang et al. 2010)

10 $$\begin{aligned} E^R(\phi , \varvec{\nabla } \phi ) = \mu \, \textrm{A}(\phi , \varvec{\nabla } \phi ) + \xi P(\varvec{\nabla } \phi ) \, , \end{aligned}$$

which controls the smoothness of the object boundary $\Gamma$ by multiplying its surface area $\textrm{A}$ with a penalty coefficient $\mu$, with $\textrm{A}$ given by

11 $$\begin{aligned} \textrm{A}(\phi , \varvec{\nabla } \phi ) = \int _{\Omega } \left| \varvec{\nabla } H_{\epsilon }\left( \phi (\textbf{x}) \right) \right| \, \textrm{d}\Omega = \int _{\Omega } \delta _{\epsilon } \left( \phi (\textbf{x})\right) \left| \varvec{\nabla } \phi (\textbf{x}) \right| \, \textrm{d}\Omega \, , \end{aligned}$$

where $\varvec{\nabla }$ represents the gradient operator and |...| denotes the length of the corresponding vector. Additionally, $\delta _{\epsilon }(\phi )$ is a regularized Dirac delta function, which follows from Eq.(6) as

12 $$\begin{aligned} \delta_{\epsilon }(\phi ) \, = \, \frac{\textrm{d} \, H_{\epsilon }(\phi )}{\textrm{d} \phi } \, = \, \frac{\epsilon }{\pi \left( \epsilon ^2+\phi ^2 \right) } \, . \end{aligned}$$

Further, the penalty function $P(\varvec{\nabla } \phi )$ in Eq.(10) reads

13 $$\begin{aligned} \textrm{P}(\varvec{\nabla } \phi ) = \frac{1}{2}\int _{\Omega } \left( \left| \varvec{\nabla } \left( \phi (\textbf{x}) \right) \right| \, - \, 1 \right) ^2 \textrm{d}\Omega \, , \end{aligned}$$

where its relative contribution to the total energy functional *E* in Eq. (3) is set by the penalty coefficient $\xi$. The penalty function enforces the level set function $\phi$ to remain close to a signed distance function during its evolution, so that a (time-consuming) reinitialization of the model equations can be avoided, see Wang et al. (2010) for more details.

### Evolution of level set function and boundary conditions

When the evolving phase boundary $\Gamma$ becomes stationary by reaching the desired image object boundary in the domain $\Omega$, the energy functional *E* presented by Eq. (3), for a given set of average intensities {$c_1,c_2,d_1,d_2$}, has been minimized with respect to the level set function $\phi$, as formalized by (Chan and Vese 1999, 2001; Wang et al. 2010)

14 $$\begin{aligned} \underset{\phi }{\text {min}} \, \, E \, \left( \phi , \varvec{\nabla } \phi \right) \, . \end{aligned}$$

The evolution of the level set function is obtained from an incremental update procedure, where, from the value $\phi _n$ at a certain incremental step *n*, the new value $\phi _{n+1}$ at the subsequent incremental step $n+1$ is determined by

15 $$\begin{aligned} \phi _{n+1} = \phi _n + \Delta \phi _{n+1} \, , \end{aligned}$$

in which $\Delta \phi _{n+1}$ is the actual, incremental change of the level set function. The discretized evolution equation for the level set function can be deduced from a variational framework, which starts by incorporating the discrete variables in Eq. (15) in the minimization condition given by Eq. (14), and reformulating this condition as

16 $$\begin{aligned} \delta \, E_{n+1}^{*} \! \left( \phi _{n+1}, \varvec{\nabla } \phi _{n+1}, \Delta \phi _{n+1} \right) \, = \, \delta \! \int _{\Omega } \mathcal {E}_{n+1}^{*} \! \left( \textbf{x}, \, \phi _{n+1}(\textbf{x}), \, \varvec{\nabla } \phi _{n+1} (\textbf{x}), \, \Delta \phi _{n+1} (\textbf{x}) \right) \textrm{d}\Omega \, = \, 0 \, , \end{aligned}$$

whereby $\delta$ denotes a small change (or variation) and $\mathcal {E}_{n+1}^{*}$ is the energy density associated to the specific form of the energy $E_{n+1}^{*}$, which will be defined below. Equation (16) can be further elaborated as

17 $$\begin{aligned} \int _{\Omega } \left( \left( \frac{\partial \mathcal {E}_{n+1}^{*}}{\partial \phi _{n+1}} \, + \, \frac{\partial \mathcal {E}_{n+1}^{*}}{\partial (\Delta \phi _{n+1})} \frac{\partial (\Delta \phi _{n+1})}{\partial \phi _{n+1}} \right) \delta \phi _{n+1} \, + \, \frac{\partial \mathcal {E}_{n+1}^{*}}{\partial \left( \varvec{\nabla } \phi _{n+1} \right) } \, \, \delta \left( \varvec{\nabla } \phi _{n+1} \right) \right) \textrm{d} \Omega = 0 \, , \end{aligned}$$

which, after applying integration by parts, and using the fact that $\partial (\Delta \phi _{n+1})/\partial \phi _{n+1}=1$, see Eq.(15), results in the Euler-Lagrange equation

18 $$\begin{aligned} \frac{\partial \mathcal {E}_{n+1}^{*}}{\partial \phi _{n+1}} \, + \, \frac{\partial \mathcal {E}_{n+1}^{*}}{\partial (\Delta \phi _{n+1})} \, - \, \varvec{\nabla } \cdot \left( \frac{\partial \mathcal {E}_{n+1}^{*}}{\partial \left( \varvec{\nabla } \phi _{n+1} \right) } \right) \, = \, 0 \, \qquad \text{ in } \qquad \Omega \, , \end{aligned}$$

and the boundary conditions

19 $$\begin{aligned} \begin{array}{lclcl} \vspace*{2mm} \displaystyle { \frac{\partial \mathcal {E}_{n+1}^{*}}{\partial \left( \varvec{\nabla } \phi _{n+1} \right) } \cdot \varvec{n}} \, = \, 0 & \qquad \text{ on } & \qquad \partial \Omega _t \, , \qquad \text{ and } \\ \phi _{n+1} = \hat{\phi }_{n+1} & \qquad \text{ on } & \qquad \partial \Omega _e \, , \qquad \text{ with } \qquad \partial \Omega = \partial \Omega _t \cup \partial \Omega _e \, , \end{array} \end{aligned}$$

where $\varvec{n}$ is the unit vector normal to the domain boundary $\partial \Omega _t$, and $\hat{\phi }_{n+1}$ is the value of the level set function prescribed at the adjoint domain boundary $\partial \Omega _e$. In the above expressions, the centred dot indicates an inner product between two vectors. The incremental update procedure has converged when the level set function becomes stationary, i.e., $\phi _{n+1} = \phi _n$ in the domain $\Omega$, which, from Eq. (15), means that the criterion $\Delta \phi _{n+1}=0$ has been reached everywhere in $\Omega$ within a small, prescribed tolerance. The convergence criterion $\Delta \phi _{n+1}=0$ is combined with the minimization problem given by Eq. (14) through expressing the energy functional $E_{n+1}^{*}$ in Eq. (16) in terms of $E_{n+1}$ as

20 $$\begin{aligned} E_{n+1}^{*} \! \left( \phi _{n+1}, \varvec{\nabla } \phi _{n+1}, \Delta \phi _{n+1} \right) \, = \, E_{n+1} \left( \phi _{n+1}, \varvec{\nabla } \phi _{n+1} \right) \, + \, \lambda \int _{\Omega } \frac{1}{2} \left( \Delta \phi _{n+1} (\textbf{x}) \right) ^2 \textrm{d} \Omega \, , \end{aligned}$$

in which $\lambda$ is a penalty coefficient. Note from Eq. (20) that $E_{n+1}^{*} \approx E_{n+1}$ when the update procedure converges at increment $n+1$. Inserting the definition for $E_{n+1}$ (as presented in Sect. Local Chan-Vese energy functional) into Eq. (20), and combining the result with Eqs. (16)—(18), specifies the Euler-Lagrange equation, Eq. (18), into the following evolution equation

21 $$\begin{aligned} \begin{aligned} \lambda \, \Delta \phi _{n+1}&= \, \delta _\epsilon \! \left( \phi _{n+1} \right) \left( \alpha \left( I - c_2 \right) ^2 \, - \, \alpha \left( I - c_1 \right) ^2 \, + \, \beta \left( g_k*I \, - \, I \, - \, d_2 \right) ^2 \right. \\&\left. \, - \, \beta \left( g_k*I \, - \, I \, - \, d_1 \right) ^2 \right) \\& + \, \mu \left( \delta _\epsilon \! \left( \phi _{n+1} \right) \, \varvec{\nabla } \cdot \left( \frac{ \varvec{\nabla } \phi _{n+1} }{ \left| \varvec{\nabla } \phi _{n+1} \right| }\right) \, + \, \delta _{\epsilon }'\left( \phi _{n+1} \right) \left| \varvec{\nabla } \phi _{n+1} \right| \right) \\&\, + \, \xi \left( \nabla ^2 \phi _{n+1} - \varvec{\nabla } \cdot \left( \frac{\varvec{\nabla } \phi _{n+1}}{ \left| \varvec{\nabla } \phi _{n+1} \right| } \right) \right) \qquad \qquad \qquad \text{ in } \qquad \Omega \, , \end{aligned} \end{aligned}$$

with $\nabla ^2 = \varvec{\nabla } \cdot \varvec{\nabla }$ the Laplace operator and the derivative $\delta '_{\epsilon }(\phi )$ of the regularized Dirac delta function obtained from Eq. (12) as

22 $$\begin{aligned} \delta '_{\epsilon }(\phi ) \, = \, \frac{\textrm{d} \, \delta _{\epsilon }(\phi )}{\textrm{d} \phi } \, = \, \frac{-2 \, \epsilon \, \phi }{\pi ^2 \left( \epsilon ^2 \, + \, \phi ^2 \right) ^2} \, . \end{aligned}$$

Further, the natural boundary condition given by Eq. (19)_1_ specifies into

23 $$\begin{aligned} \left( \mu \, \delta _\epsilon \! \left( \phi _{n+1} \right) \, \frac{ \varvec{\nabla } \phi _{n+1} }{ \left| \varvec{\nabla } \phi _{n+1} \right| } \, + \, \xi \left( \varvec{\nabla } \phi _{n+1} - \frac{\varvec{\nabla } \phi _{n+1}}{ \left| \varvec{\nabla } \phi _{n+1} \right| } \right) \right) \cdot \varvec{n} \, = \, 0 \, \qquad \text{ on } \qquad \partial \Omega _t \, , \end{aligned}$$

while the essential boundary condition remains unchanged, in accordance with Eq. (19)_2_. In the present work, the two phases that need to be segmented may evolve along the outer domain boundary $\partial \Omega$ during the convergence process; this makes the essential boundary condition Eq. (19)_2_, whereby the phases along the boundary are prescribed, inappropriate. The natural boundary condition given by Eq. (23) automatically allows for the evolution of the two phases along the outer domain boundary, and can be rigorously satisfied by requiring that

24 $$\begin{aligned} \varvec{\nabla } \phi _{n+1} \cdot \varvec{n} \, = \, 0 \, \qquad \text{ on } \qquad \partial \Omega \, . \end{aligned}$$

This boundary condition has been also applied in the works of Chan and Vese (1999, 2001); Wang et al. (2010) that provide the basic formulation of the (Local) Chan-Vese model.

In order to incrementally update the level set function $\phi$ from the evolution equation Eq. (21), the term $\varvec{\nabla } \cdot \left( {\varvec{\nabla } \phi }/{|\varvec{\nabla } \phi |}\right)$, which is known as the curvature $\kappa$, is developed in *x*-, *y*- and *z*-components as

25 $$\begin{aligned} \begin{aligned} \kappa = \, &\varvec{\nabla } \cdot \left( \frac{\varvec{\nabla }\phi }{|\varvec{\nabla }\phi |} \right) = \left( \rule{0mm}{5mm} \phi _{,xx} (\phi _{,y})^2 + \phi _{,xx} (\phi _{,z})^2 + \phi _{,yy} (\phi _{,x})^2 + \phi _{,yy} (\phi _{,z})^2 \right. \\& \qquad \qquad + \phi _{,zz} (\phi _{,x})^2 + \phi _{,zz} (\phi _{,y})^2 \left. \rule{0mm}{5mm} - 2\phi _{,xy} \phi _{,x} \phi _{,y} - 2\phi _{,xz} \phi _{,x} \phi _{,z} - 2\phi _{,yz} \phi _{,y} \phi _{,z} \right) \\& \qquad \qquad {\, \times \left( \rule{0mm}{5mm} (\phi _{,x})^2 + (\phi _{,y})^2 + (\phi _{,z}^2 )\right) ^{-3/2}} \, , \end{aligned} \end{aligned}$$

where the subscript , *m* denotes the partial derivative in the *m*-direction. In addition, the terms $\nabla ^2 \phi$ and $|\varvec{\nabla } \phi |$ appearing in Eq. (21) in component form read

26 $$\begin{aligned} \begin{array}{lclcl} \nabla ^2 \phi & = & \phi _{,xx} + \phi _{,yy} + \phi _{,zz} \, , \\ | \varvec{\nabla } \phi | & = & \left( \left( \phi _{,x} \right) ^2 \, + \, \left( \phi _{,y} \right) ^2 \, + \, \left( \phi _{,z} \right) ^2 \right) ^{1/2} \, . \end{array} \end{aligned}$$

In each voxel (*i*, *j*, *k*), the spatial derivatives in Eqs. (25) and (26) are computed from the level set values in neighbouring voxels by adopting a finite difference scheme:

27 $$\begin{aligned} \begin{array}{lclcl} \vspace*{2mm} \phi _{,x}^{(i,j,k)} & = & \displaystyle { \frac{1}{2h} \left( \phi ^{(i+1,j,k)}-\phi ^{(i-1,j,k)} \right) \, , } \\ \vspace*{2mm} \phi _{,y}^{(i,j,k)} & = & \displaystyle { \frac{1}{2h} \left( \phi ^{(i,j+1,k)}-\phi ^{(i,j-1,k}) \right) \, , } \\ \vspace*{2mm} \phi _{,z}^{(i,j,k)} & = & \displaystyle { \frac{1}{2h} \left( \phi ^{(i,j,k+1)}-\phi ^{(i,j,k-1)} \right) \, , } \\ \vspace*{2mm} \phi _{,xx}^{(i,j,k)} & = & \displaystyle { \frac{1}{h^2}(\phi ^{(i+1,j,k)} - 2\phi ^{(i,j,k)} + \phi ^{(i-1,j,k)}) \, , } \\ \vspace*{2mm} \phi _{,yy}^{(i,j,k)} & = & \displaystyle { \frac{1}{h^2} \left( \phi ^{(i,j+1,k)} - 2\phi ^{(i,j,k)} + \phi ^{(i,j-1,k)} \right) \, , } \\ \vspace*{2mm} \phi _{,zz}^{(i,j,k)} & = & \displaystyle { \frac{1}{h^2} \left( \phi ^{(i,j,k+1)} - 2\phi ^{(i,j,k)} + \phi ^{(i,j,k-1)} \right) \, , } \\ \vspace*{2mm} \phi _{,xy}^{(i,j,k)} & = & \displaystyle { \frac{1}{4h^2}\left( \phi ^{(i+1,j+1,k)} - \phi ^{(i+1,j-1,k)} - \phi ^{(i-1,j+1,k)} + \phi ^{(i-1,j-1,k)} \right) \, , } \\ \vspace*{2mm} \phi _{,xz}^{(i,j,k)} & = & \displaystyle { \frac{1}{4h^2} \left( \phi ^{(i+1,j,k+1)} - \phi ^{(i+1,j,k-1)} - \phi ^{(i-1,j,k+1)} + \phi ^{(i-1,j,k-1)} \right) \, , } \\ \phi _{,yz}^{(i,j,k)} & = & \displaystyle { \frac{1}{4h^2} \left( \phi ^{(i,j+1,k+1)} - \phi ^{(i,j+1,k-1)} - \phi ^{(i,j-1,k+1)} + \phi ^{(i,j-1,k-1)} \right) \, , } \end{array} \end{aligned}$$

with *h* the edge length of a cuboidal voxel (which has been scaled to unity). The solution of Eq. (21), in principle, requires the application of an implicit, iterative update scheme. However, in this work a more straightforward, explicit update scheme is applied, for which, in the right-hand side of Eq. (21), the level set evaluations at the current increment $n+1$ are approximated by those at the *previous* increment *n*, i.e.,

28 $$\begin{aligned} \begin{aligned} \lambda \, \Delta \phi _{n+1}&= \, \delta _\epsilon \! \left( \phi _{n} \right) \left( \alpha \left( I - c_2 \right) ^2 \, - \, \alpha \left( I - c_1 \right) ^2 \, + \, \beta \left( g_k*I \, - \, I \, - \, d_2 \right) ^2 \, \right. \\&\left. \, - \, \beta \left( g_k*I \, - \, I \, - \, d_1 \right) ^2 \right) \, + \, \mu \left( \delta _\epsilon \! \left( \phi _{n} \right) \, \varvec{\nabla } \cdot \left( \frac{ \varvec{\nabla } \phi _{n} }{ \left| \varvec{\nabla } \phi _{n} \right| }\right) \, + \, \delta _{\epsilon }'\left( \phi _{n} \right) \left| \varvec{\nabla } \phi _{n} \right| \right) \, \\& + \, \xi \left( \nabla ^2 \phi _{n} - \varvec{\nabla } \cdot \left( \frac{\varvec{\nabla } \phi _{n}}{ \left| \varvec{\nabla } \phi _{n} \right| } \right) \right) \qquad \qquad \qquad \text{ in } \qquad \Omega \, . \end{aligned} \end{aligned}$$

When substituting the time and spatial discretizations given by Eqs. (15), (25), (26) and (27) in Eq. (28), the updated value of the level set function $\phi _{n+1}^{(i,j,k)}$ in voxel (*i*, *j*, *k*) is obtained as

29 $$\begin{aligned} \phi ^{(i,j,k)}_{n+1}&= \, \phi ^{(i,j,k)}_{n} \, + \, \delta _\epsilon \! \left( \phi _{n}^{(i,j,k)} \right) \left[ \frac{\alpha }{\lambda } \left( I^{(i,j,k)} - c_2 \right) ^2 \, - \, \frac{\alpha }{\lambda } \left( I^{(i,j,k)} - c_1 \right) ^2 \right. \\&\, + \, \frac{\beta }{\lambda } \left( \left( g_k*I\right) ^{(i,j,k)} \, - \, I^{(i,j,k)} - \, d_2 \right) ^2 \left. \, - \, \frac{\beta }{\lambda } \left( \left( g_k*I\right) ^{(i,j,k)} \, - \, I^{(i,j,k)} \, - \, d_1 \right) ^2 \right] \\& + \,\frac{\mu }{\lambda } \left[ \delta _\epsilon \left( \phi _{n}^{(i,j,k)} \right) \kappa _{n}^{(i,j,k)} \right. \left. \, + \, \delta _{\epsilon }' \! \left( \phi _{n}^{(i,j,k)} \right) \left| \varvec{\nabla } \phi _{n}^{(i,j,k)} \right| \right] \, + \, \frac{\xi }{\lambda } \left[ \nabla ^2 \phi _{n}^{(i,j,k)} - \kappa _{n}^{(i,j,k)} \right] \, . \end{aligned}$$

As explained, the incremental update procedure is terminated once the convergence criterion $\Delta \phi _{n+1}=0$ is satisfied within a small, prescribed tolerance $\epsilon _{tol}$, which, for the overall domain $\Omega$ composed of $N_x \times N_y \times N_z$ voxels, is conveniently expressed in terms of an $L_2$-norm as

30 $$\begin{aligned} \left( \sum _{i=1}^{N_x} \sum _{j=1}^{N_y} \sum _{k=1}^{N_z} \left( \phi ^{(i,j,k)}_{n+1} - \phi ^{(i,j,k)}_{n} \right) ^2 \right) ^{1/2} \, < \, \epsilon _{tol} \, . \end{aligned}$$

In order to effectively and rigorously reach the above convergence criterion, in Eq. (29) the penalty coefficients $\mu$ and $\xi$ at the onset of each new increment $n+1$ are multiplied by a factor equal to the inverse of the maximum over all voxels (*i*, *j*, *k*) of the magnitude characterizing the actual value of the corresponding penalty function, which, for both $\mu$ and $\xi$, is given by

31 $$\begin{aligned} \left( \underset{(i,j,k)}{\text{ max }} \left( \left| \varvec{\nabla } \phi _n^{(i,j,k)} \right| \right) \right) ^{-1} \, , \end{aligned}$$

see Eqs. (11) and (13). Since the updated value of the penalty function associated to the penalty coefficient $\lambda$ is determined by the new value $\phi _{n+1}$ of the level set function (which at the onset of the increment is unknown), see Eqs. (20) and (15), for simplicity the value of $\lambda$ is kept constant during the incremental update procedure.

In the above model formulation, the discretized evolution equation and the corresponding boundary conditions of the image segmentation method have been rigorously derived from a variational framework, and contain specific features that are different, or absent, in the original model formulations presented in Chan and Vese (1999, 2001); Wang et al. (2010). These features concern (i) the presence of the term $\delta _{\epsilon }' \! \left( \phi _{n}^{(i,j,k)} \right) \left| \varvec{\nabla } \phi _{n}^{(i,j,k)} \right|$ in the discretized evolution equation, Eq.(29), (ii) the derivation of the boundary conditions in their general form, Eqs. (23) and (19)_2_, (iii) the discretized evolution equation, Eq. (29), being time-independent (as it formally must be), iv) the scaling of penalty coefficients in the energy functional via Eq. (31), and (v) the extension of the model formulation (and the corresponding numerical implementation) from 2D to 3D image segmentation. Although these features positively affect the generality, robustness and convergence speed of the image segmentation approach, their influence on the final computational result is expected to be moderate to small, as the computational result is mainly determined by the specific definition of the energy functional, Eq. (3), which is the same as in Wang et al. (2010).

### Skeleton-based image segmentation into axial cells and ray parenchyma cells

**Fig. 2:**
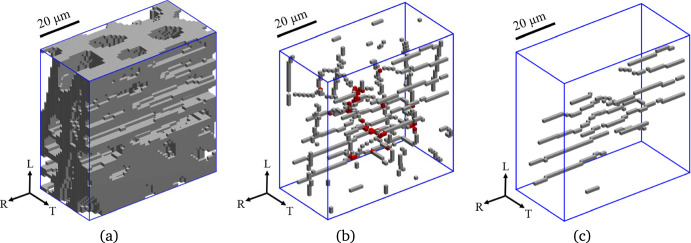
Stepwise procedure of a skeleton-based image segmentation into axial cells and ray parenchyma cells. **a** Binary image of the cellular structure of a 3D oak wood sample, where the grey objects designate the cell walls and the space in between is occupied by the cell cavities. **b** Skeleton constructed from the medial axes of *both* the axial cells and the ray parenchyma cells. The interconnection between axial cells and ray cells are indicated by the red voxels. **c** Skeleton constructed from the medial axes of *only* the ray parenchyma cells (colour figure online)

With the level set-based image segmentation model presented in Sects. "Level set function" to "Evolution of level set function and boundary conditions", a three-dimensional oak wood micro-structure can be segmented into two different objects, which are the cell walls and the cell cavities (i.e., lumen, vessels). The cell wall objects can be further segmented into more detailed objects that are representative of the cell types associated to different material directions of wood. Oak wood can be considered as an orthotropic material, where the three principal material directions correspond with the longitudinal (L) direction of the tree stem, and the radial (R) and tangential (T) directions of the seasonal growth rings that appear as concentric layers in the cross-section of the stem. The additional image segmentation step into cell types associated to specific material directions is performed by starting from the binary image of the cellular structure of a 3D oak wood sample shown in Fig. 2a, which has been obtained by applying the image segmentation method formulated in Sects. "Local Chan-Vese energy functional" and "Evolution of level set function and boundary conditions" to a $\mu$CT image of the oak micro-structure. The binary image has been straightforwardly constructed from the values of the level set function $\phi (\textbf{x})$, where the grey objects designating the cell wall material correspond to a positive value of $\phi (\textbf{x})$, and the space in between, which is occupied by the cell cavities, corresponds to a negative value of $\phi (\textbf{x})$. The cell walls relate to the axial cells and the ray parenchyma cells oriented in, respectively, the longitudinal and radial material directions of oak wood. The axial cells include fibers, axial parenchyma, earlywood vessels and latewood vessels, and will be analyzed in more detail in Sect. "Detailed features of cell morphology".

In order to univocally distinguish the axial cells from the ray parenchyma cells, the cell cavity objects are converted to their medial axes of a single voxel wide to obtain their skeleton. This process is known as “skeletonization” (Saha et al. 2016) and simplifies the micro-structural geometry while keeping the orientation and distribution of specific objects unaltered. Figure 2b displays the skeleton of the cell cavities, which coalesces with i) the medial axes of *axial cells* (mainly oriented in the longitudinal direction), ii) the medial axes of the *ray parenchyma cells* (mainly oriented in the radial direction), and iii) their interconnections (indicated by the red voxels). The interconnections need to be removed from the skeleton in order to separate the axial cells from the ray parenchyma cells. The resulting skeleton structure allows to distinguish the skeleton voxels of the axial cells from those of the ray parenchyma, with Fig. 2c illustrating, as an example, the skeleton of the segmented ray parenchyma cells. Finally, the segmentation of the cell wall voxels into axial cell wall voxels and ray cell wall voxels follows from determining to which of the two types of skeletons the perpendicular distance between a cell wall voxel and a skeleton voxel is the smallest.

### Statistical information on geometrical micro-structural features

**Fig. 3:**
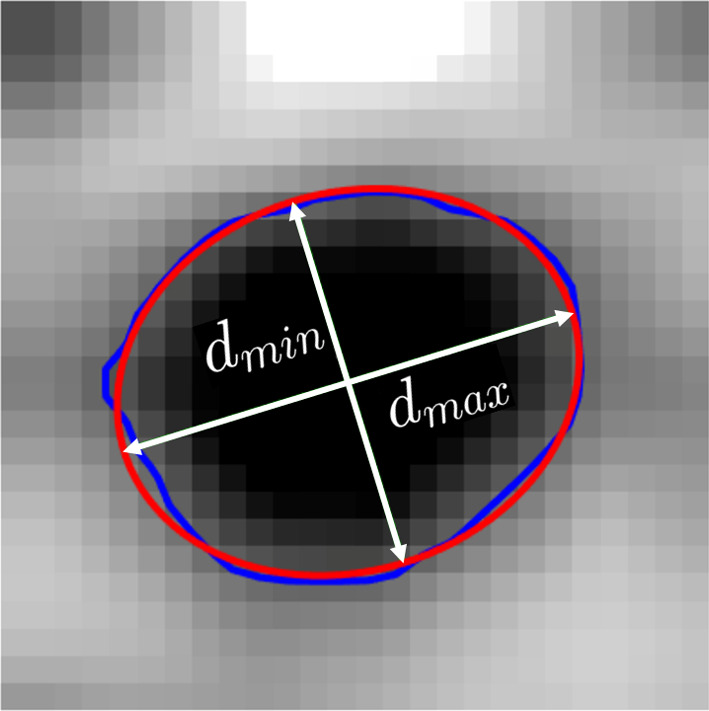
Ellipse fitting (red solid line) of the inner surface of a cell (blue solid line). The minimum diameter $d_{min}$ and maximum diameter $d_{max}$ of the ellipse are designated by the white measuring lines (colour figure online)

The above image segmentation step facilitates the collection of statistical information on geometrical features of axial cells and radial ray parenchyma cells from cross-sectional images taken across the principal material (R-T, R-L, T-L) planes of the oak micro-structure. In Sect. "Statistical information on inner cell diameters and cell wall thickness", two specific geometrical features of the axial cells and ray cells will be statistically analyzed from micro-structures of oak wood samples, which are the (i) inner dimensions of the cells and (ii) their wall thickness. For this purpose, the cross-sectional cell shapes of cells are fitted by ellipses, so that the inner dimensions of a cell can be simply expressed in terms of the minimum and maximum diameters of the cell. The elliptical cell shape is calculated by applying a standard ellipse fitting method, which, on average, captures the cross-sectional geometry of the cells reasonably well; an illustrative example of the ellipse fitting procedure is presented in Fig. 3, where the blue solid line represents the inner circumference of the cell, as determined by the zero value of the converged level set function at the spatial coordinates $\textbf{x}$ of the inner surface, i.e., $\phi _{n+1}(\textbf{x})=0$, see also Eq. (2), and the red solid line reflects the fitted ellipse, with the white measuring lines indicating its minimum and maximum diameters, $d_{min}$ and $d_{max}$.

**Fig. 4:**
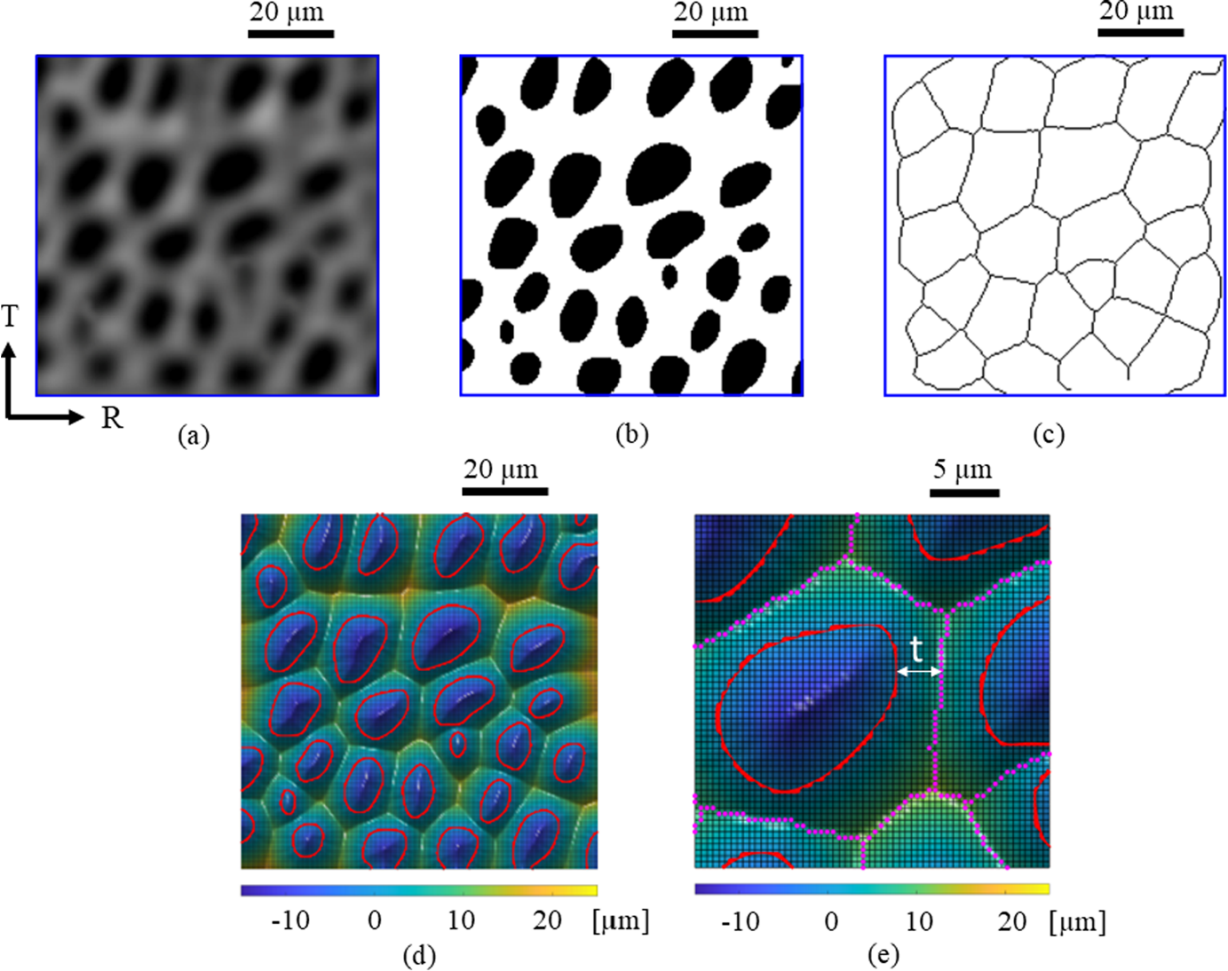
Stepwise procedure for computing the local thickness of axial cell walls. **a** Greyscale image of a wood sample cross-section in the R-T plane, obtained by $\mu$CT scanning. **b** Corresponding binary image obtained by the image segmentation method. **c** Skeleton represented by the medial axes of the cell walls of axial cells. **d** Contour plot of the values of the converged level set function $\phi _{n+1}(\textbf{x})$, with the inner surfaces of axial cells ($\phi _{n+1}(\textbf{x})=0$) indicated by a red solid line. **e** Detailed view of the cell wall geometry, with the inner surface of cell walls indicated by a red solid line, and the skeleton of the cell walls designated by the purple dotted line, where the (small) dots indicate the specific locations at which the local cell wall thickness *t* is computed. For clarification, the local cell wall thickness *t* is denoted at one arbitrary location (colour figure online)

The local cell wall thickness of the axial and ray cells is calculated from cross-sectional images of segmented wood micro-structures. Due to the irregular surface geometry and connectivity of cells, taking a measurement perpendicular to the medial axis defining the skeleton does not always provide a representative value of the local cell wall thickness. The local cell wall thickness is therefore characterized by an *alternative measure*, which equals the distance between a skeleton material point and the nearest point at the inner surface of one of the two most adjacent cells. Note from the definition of the medial axis that it makes no difference which of the two adjacent cells is selected, since they result in the same value. Figure 4 summarizes this procedure, which starts from constructing a binary image of the two segmented phases (i.e., the cell walls and cell cavities) from the greyscale image of a wood sample cross-section, taken here in the R-T plane, see Fig. 4a and b, from which the cell wall skeleton of the axial cells is constructed, see Fig. 4c, which is subsequently used for computing the local cell wall thickness *t* from the shortest distance between a skeleton material point and the cell wall boundary, see Fig. 4d and the detailed view in Fig. 4e. The locations of the cell wall boundaries are characterized by a zero value of the converged level set function, $\phi _{n+1}(\textbf{x})=0$, and are indicated in Fig. 4d and e by a red solid line. The cell wall skeleton is designated by a purple dotted line in the detailed view of the cell wall geometry in Fig. 4(e), where the (small) dots indicate the locations at which the local cell wall thickness *t* is calculated. For clarification, the local cell wall thickness *t* is denoted in Fig. 4e at one arbitrary location; it approximately equals the sum of the thicknesses of the three secondary (S1, S2 and S3) cell wall layers and half of the thickness of the compound middle lamella (CML), layer, see e.g. Scheperboer et al. (2019); Livani et al. (2023) for the specific definitions of these layers.

With the above procedure, the inner cell diameters $d_{max}$ and $d_{min}$ and the local cell wall thicknesses *t* are determined from a number of cross-sectional images taken at a regular mutual distance across a specific principal material plane of the micro-structural oak sample. The number of cross-sectional images should be sufficiently large to adequately account for statistical variations in the geometrical parameters in the direction perpendicular to the principal material plane considered. From the geometrical data, density histograms are next constructed that show the proportional distributions of the parameter values across the overall micro-structural sample, see Sect. "Statistical information on inner cell diameters and cell wall thickness" for the results.

## Performance and results of image segmentation method

In this section the performance and results of the image segmentation method are analyzed. Section "X-ray micro-computed tomography experiments" discusses the X-ray micro-computed tomography $$\mbox{$(\mu\text{CT})$}$$ experiments carried out for obtaining detailed microstructural images of two representative oak samples containing a single growth ring. Subsequently, the robustness and convergence speed of the image segmentation method are studied in Sect. "Robustness and convergence behaviour" by using as input a greyscale image of the micro-structure of a local latewood region within an oak wood growth ring. The incremental update procedure of the phase boundaries is performed for three different choices of the initial two-phase configuration, after which the convergence process towards the final, segmented micro-structure is analyzed. Section "Comparison with results from other image segmentation methods" assesses the accuracy of the image segmentation result through a comparison with the results obtained by two other image segmentation methods presented in the literature. In Sect. "Detailed features of cell morphology" specific morphological features of the segmented latewood micro-structures are visualized in detail. The computational cost of the image segmentation method is evaluated in Sect. "Comparison of computational cost on CPU and GPU hardware" by comparing its performance on CPU and GPU hardware. Finally, in Sect. "Statistical information on inner cell diameters and cell wall thickness"" statistical information on the inner cell dimensions and the cell wall thickness is deduced from the segmentation result of the two growth ring samples.

The results presented in this section are obtained by implementing the image segmentation model using the MATLAB numerical computing platform. MATLAB is chosen for its high-quality 3D visualization tools for the creation of iso-surfaces and binary images, and facilitates the skeletonization and ellipse fitting procedures applied in the analysis. In addition, MATLAB allows using a standard version of Otsu’s grey level thresholding method, which is employed in the comparison study presented in Sect. "Comparison with results from other image segmentation methods". Further, for the comparison of the computational cost on CPU and GPU hardware treated in Sect. "Comparison of computational cost on CPU and GPU hardware", the image segmentation method has been implemented in the open-source Python programming environment. The standard and optimized functions and types within the PyTorch library of Python allow to create a robust interface for seamless execution on both CPU and GPU architectures with minimal modifications of the programming script.

### X-ray micro-computed tomography experiments

Two cuboidal oak samples were analyzed using a Phoenix Nanotom S $\mu$CT instrument. The $\mu$CT instrument was equipped with a high-resolution X-ray source generating X-rays with a voltage of 60 kV and a current of 240 $\mu$A. The two samples were cut from a larger specimen along the three principal material (R-T, R-L, T-L) planes, and had approximate dimensions of $4 \times 6 \times 2$ $\hbox {mm}^3$ and $5 \times 5 \times 2$ $\hbox {mm}^3$. The two specimens were respectively provided by the Danish Agency for Culture and Palaces, Copenhagen, Denmark, and the Rijksmuseum, Amsterdam, the Netherlands, and are representative of oak wood used in historical cabinets and panel paintings. The samples were securely mounted on the rotating stage of the $\mu$CT instrument and subjected to X-rays, thus producing transmission data. The $\mu$CT scanning was performed under laboratory conditions, at a relative humidity RH $= 50 \%$ and a temperature T $= 21^{~\circ }$C, which define the reference state of the samples. The total scanning time of each wood sample was 4 h.

**Fig. 5:**
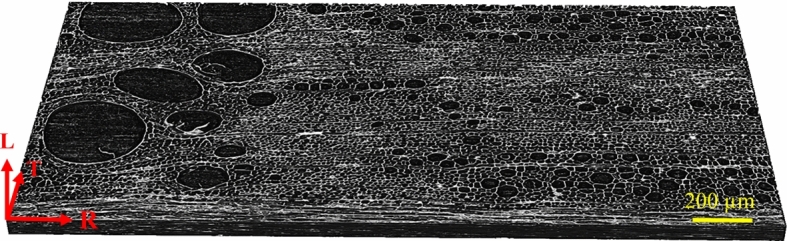
Image of an oak wood micro-structure containing a single growth ring and a horizontal band of multi-seriate rays (located at the bottom edge of the micro-structural region), as obtained from $\mu$CT scanning. The original contrast of the scanned greyscale image has been increased in this figure to enlarge the clarity of micro-structural details (colour figure online)

The 2D X-ray projection images obtained from the $\mu$CT scanning process were converted to 3D volumes using Phoenix Datos|x software. Subsequently, VGStudio MAX 2.2 was employed to refine the volumetric data by adjusting the contrast and colour thresholds. The multi-channel images constructed were next converted to greyscale images using the image processing toolbox of MATLAB, by removing the hue and saturation components while preserving the luminance. The three-dimensional greyscale images were generated using a voxel size of $1.3 \times 1.3 \times 1.3$ $\mu \hbox {m}^3$.

In order to carry out a statistical analysis on the geometrical features of a segmented oak micro-structure, from each of the two scanning results a representative meso-structural region was identified that contains a single growth ring and a horizontal band of multi-seriate rays that runs along the bottom edge of the micro-structural region, see Fig. 5. Note that the characteristics of the micro-structural samples may depend on density variations across growth rings. This aspect has been accounted for in Livani et al. (2023) for the determination of the effective hygro-mechanical properties of oak wood, showing that for three separate growth ring samples with considerable differences in density, the spread in the effective properties can be on the order of magnitude of their average values. In the present work, the effect of micro-structural density variations on the analysis result is not studied in further detail, as the main goal here is to assess the overall performance of the image segmentation method, which can be achieved by using two arbitrary micro-structural samples.

**Table 1:** Characteristics of two different oak samples containing a single growth ring and a band of multi-seriate rays, as determined from $\mu$CT images. The abbreviation “EW” refers to “earlywood”

Sample	Porosity	Density	Width	Height	Thickness	Ray volume fraction	Width of EW	Vessel volume fraction in EW
	$\phi$	$\rho$	*W*	*H*	$\mathcal {T}$	$v_{\textrm{ray}}$	$W_e$	$v_{\textrm{vess,EW}}$
	[-]	[kg/$\hbox {m}^3$]	[$\mu$m]	[$\mu$m]	[$\mu$m]	[-]	[$\mu$m]	[-]
A	0.454	786	2600	1300	65	0.21	756	0.14
B	0.481	748	2600	1469	65	0.19	1021	0.16

The geometrical and physical characteristics of the two micro-structural regions from hereon referred to as samples A and B are listed in Table 1. The width *W*, height *H*, and thickness $\mathcal {T}$ represent the dimensions of a sample along the R-, T-, and L-directions, respectively. Additionally, $W_e$ reflects the width of the earlywood (EW) region, from which the width of the latewood region straightforwardly is obtained as ($W-W_e$). The porosities $\phi$ of the samples are equal to 0.454 and 0.481. Assuming a cell wall density in the dry state of $\rho _{cw} = 1440$ kg/$\hbox {m}^3$ (Kellogg and Wangaard 1969), the densities $\rho = (1-\phi )\rho _{cw}$ of the two samples become $\rho _A=786$ kg/$\hbox {m}^3$ and $\rho _B=748$ kg/$\hbox {m}^3$.

### Robustness and convergence behaviour

**Fig. 6:**
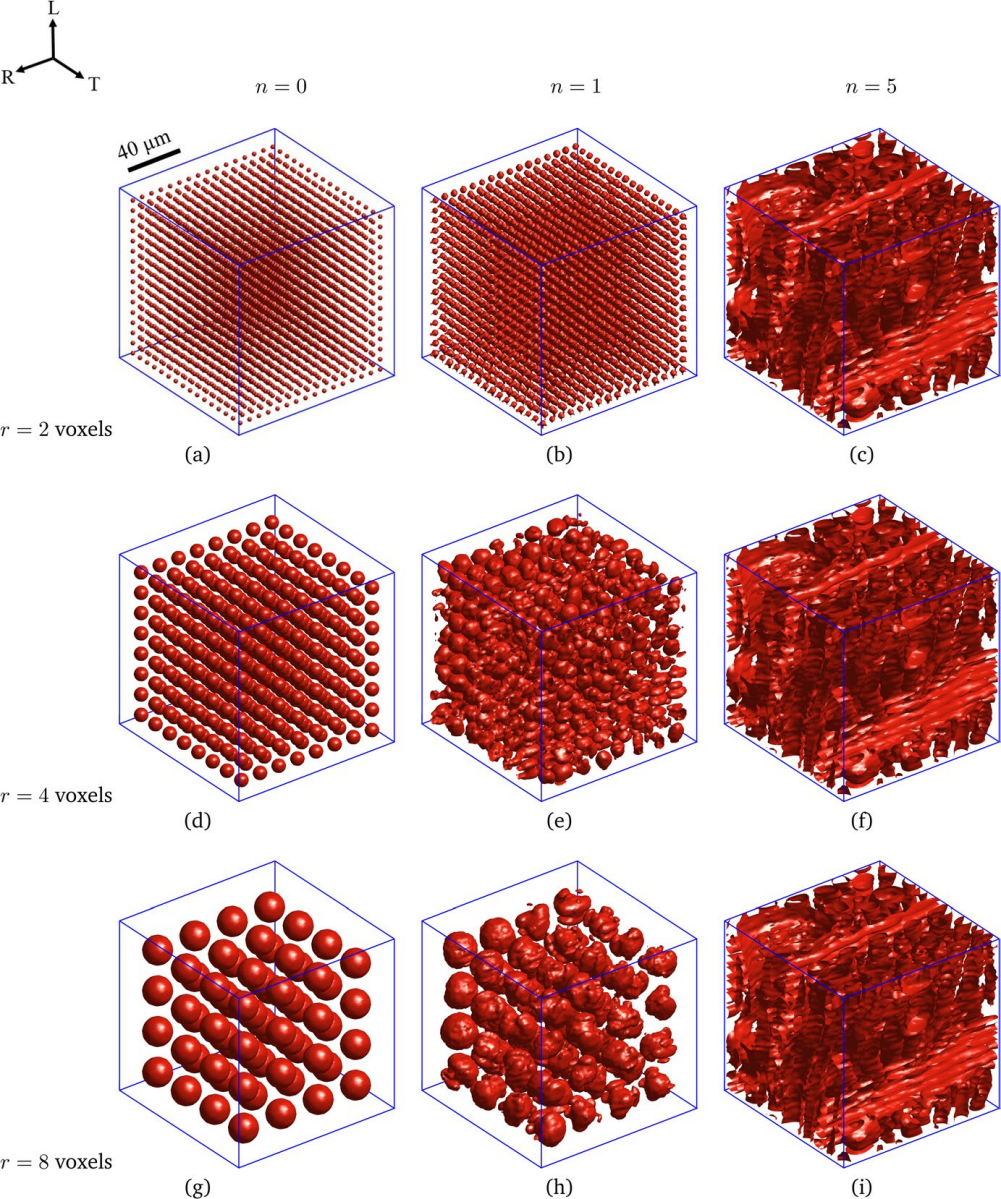
Convergence behaviour of the image segmentation process illustrated by the evolution of the phase boundaries (depicted in red) between the cell walls and the cell cavities in a *local latewood region*. Three different initial configurations ($n=0$) of uniformly distributed, spherical cell cavities are selected, as presented in the left column of figures, where the cavity radius equals $r=2$ voxels (**a**), $r=4$ voxels (**d**) and $r=8$ voxels (**g**). The corresponding segmentation result after the first incremental step, $n=1$, of the level set update procedure, as captured by Eq. (28), is shown in the middle column of figures, (**b**, **e**, **h**), while the corresponding final, converged segmentation result after $n=5$ incremental steps is illustrated in the right column of figures, (**c**, **f**, **i**) (colour figure online)

Before performing a statistical analysis on the geometrical micro-structural features of samples A and B, the robustness and convergence behaviour of the image segmentation method are studied by employing as input a greyscale image of a *local latewood region* within an oak wood growth ring. The latewood region is cube-shaped and has a size of $130 \times 130 \times 130$ $\mu \hbox {m}^3$, in correspondence with an image composed of 100 voxels in each direction. Figure 6 shows the convergence behaviour of the image segmentation process by depicting (in red) the evolution of the phase boundaries between the cell walls and the cell cavities in the latewood region, as defined by a zero value of the level set function, $\phi _{n}(\textbf{x})=0$. The image segmentation process has been performed by adopting the following (initial) values for the parameters in Eqs. (21) and (22) defining the Local Chan-Vese energy functional: $\alpha = 1$, $\beta =1$, $\mu =0.1$, $\xi =1$, $\lambda =2$ and $\epsilon =1$. Note here that the penalty coefficients $\mu$ and $\xi$ are updated at each increment by applying the scaling factor in Eq. (31). The evolution of the phase boundaries is shown for three different initial configurations ($n=0$) of uniformly distributed, spherical cell cavities presented in the left column of figures, of which the cavity radius equals $r=2$ voxels (Fig. 6a), $r=4$ voxels (Fig. 6d) and $r=8$ voxels (Fig. 6g). The corresponding segmentation result after the first incremental step, $n=1$, of the level set update procedure, as captured by Eq. (28), is shown in the middle column of figures, i.e., Fig. 6b, e and h, while the corresponding final, converged segmentation result after $n=5$ incremental steps is illustrated in the right column of figures, i.e., Fig. 6c, f and i. From the morphological differences in the configurations depicted in the middle column of figures, it can be concluded that the convergence path for the three initial configurations clearly is dependent on the chosen initial configuration. Nevertheless, the final result displayed in the right column of figures is almost identical for the three cases, from which it may be concluded that the image segmentation method behaves robustly by providing a converged segmentation result that is unique and independent of the chosen initial configuration.

**Fig. 7:**
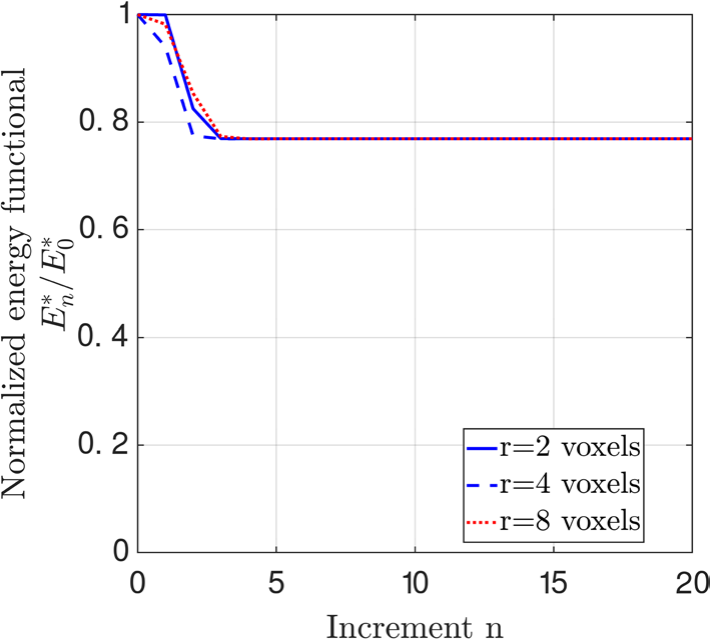
Convergence behaviour of the image segmentation process: Energy functional $E_{n}^{*}$, Eq. (20), as a function of the number of increments *n* of the image segmentation process, for different initial configurations of uniformly distributed spherical cell cavities with a cavity radius of $r=2$ voxels (blue solid line), $r=4$ voxels (blue dashed line), and $r=8$ voxels (red dotted line), as depicted in the left column of figures in Fig. 6 (colour figure online)

The specific convergence behaviour of the image segmentation method towards the final result can be studied by plotting the value of the energy functional $E_{n}^{*}$—which via Eq. (20) is used as the objective function in the minimization procedure Eq. (14)—as a function of the number of increments *n*. The convergence trend is shown in Fig. 7 for the different initial configurations of uniformly distributed, spherical cell cavities with a cavity radius of $r=2$ voxels (blue solid line), $r=4$ voxels (blue dashed line), and $r=8$ voxels (red dotted line), whereby the energy functional $E_{n}^{*}$ has been normalized by its initial value, $E_{0}^{*}$. It is seen that the monotonically decreasing convergence trend for the three initial configurations is somewhat different, but that for all three cases the converged segmentation result is reached very fast, i.e., at $n=2$ increments (*r *= 4 voxels) or $n=3$ increments (*r *= 2 voxels and *r *= 8 voxels), after which the energy functional preserves its minimum value at subsequent increments. It may thus be concluded that the image segmentation algorithm converges very efficiently, especially considering that the initial two-phase configurations chosen in the examples are rather different from the final, converged configuration, see Fig. 6.

### Comparison with results from other image segmentation methods

The relative accuracy of the present level-set image segmentation method is assessed by comparing its results to those obtained with the well-known grey level thresholding method of Otsu (Otsu 1979), and the results acquired by level-set image segmentation based on the Global Chan-Vese energy functional (Chan and Vese 1999, 2001). The segmentation results from Otsu’s grey level thresholding method are computed through utilizing the standard algorithm available in the commercial software program MATLAB, which, by minimizing intra-class intensity variance, computes a single intensity threshold that separates the voxels composing the image into two distinct classes/phases. The phase boundaries between the two phases are next determined by computing the value of the signed distance function for each voxel, and then calculating the locations at which the signed distance function equals zero, in accordance with Eqs. (1) and (2). Additionally, the level-set image segmentation result based on the Global Chan-Vese energy functional is computed with the model formulation presented in Sect. "Level set-based image segmentation method", by setting the parameter $\beta$ in Eq. (3) to zero, i.e., the segmentation contribution related to local image information is switched off. The remaining parameter values are the same as selected for the Local Chan-Vese energy functional, as reported in Sect. "Robustness and convergence behaviour".

**Fig. 8:**
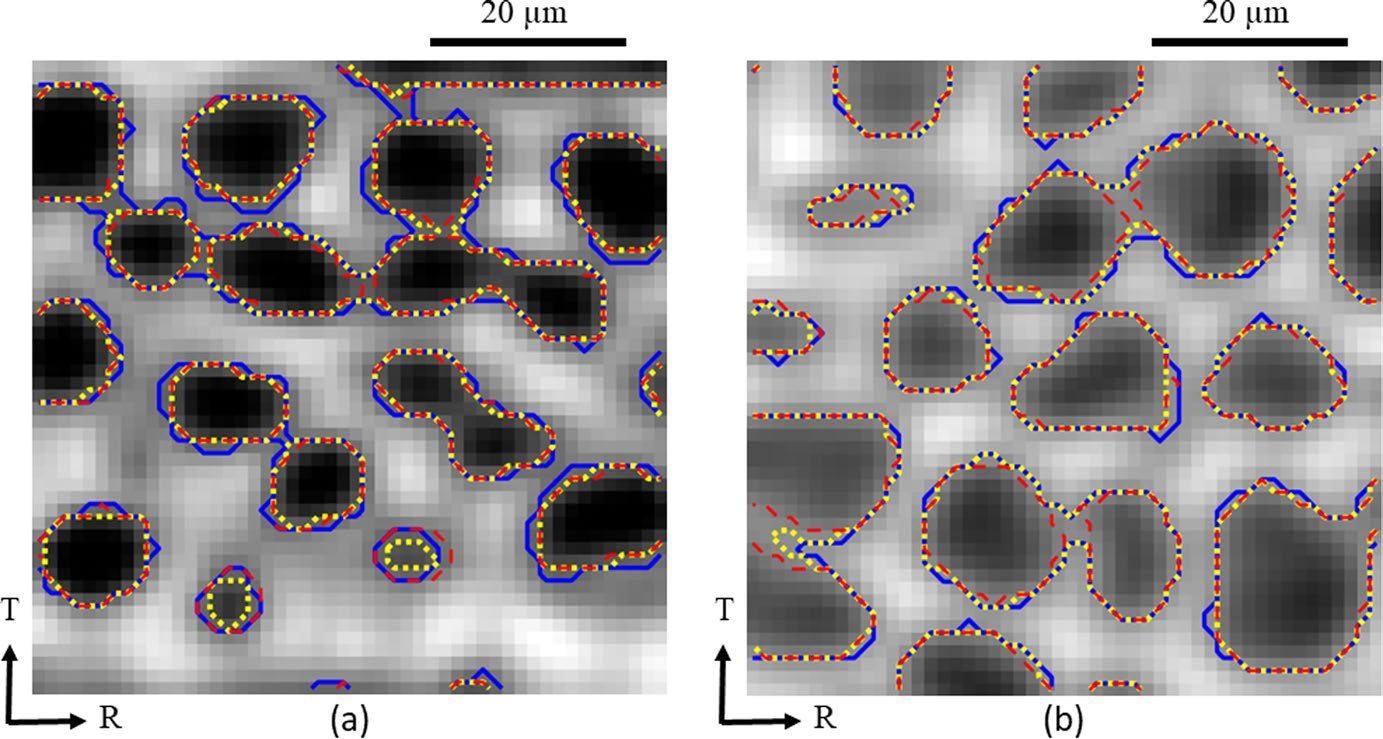
Comparison of segmentation results obtained by three different image segmentation methods in the R-T plane of a local latewood region within an oak growth ring, i.e., the Local Chan-Vese method (red dashed line), the Global Chan-Vese method (yellow dotted line), and Otsu’s grey level thresholding method (blue solid line). **a** Greyscale image with *high phase contrast* between cell cavities (black/dark grey) and cell walls (medium grey/light grey). **b** Greyscale image with *medium phase contrast* between cell cavities (dark grey/medium grey) and cell walls (medium grey/light grey). The contrast and variation in grey scale levels defining the two figures above are representative of the entire scanning volume of samples A and B (colour figure online)

The results computed by the three image segmentation methods are compared in Fig. 8 for grey scale images in the R-T plane of a local latewood region within an oak growth ring, considering images with, respectively, high phase contrast, Fig. 8a, and medium phase contrast, Fig. 8b. The choice of a region in the R-T plane is arbitrary here, and another plane could have been selected instead. The contrast and variation in grey scale levels depicted in the figures are representative of the entire scanning volume of samples A and B. The level-set image segmentation results computed from the Local and Global Chan-Vese energy functionals are indicated by the red dashed and yellow dotted lines, respectively, and the result obtained from Otsu’s grey level thresholding method is designated by the blue solid line. Figures 8a and b both show that Otsu’s thresholding method locally *overestimates* the size of cell cavity regions through the incorporation of small cell wall regions. For the image segmentation method based on the Global Chan-Vese energy functional this also occurs, but to a somewhat lesser extent. In addition, Fig. 8a illustrates that the size of relatively small cell cavity regions is *underestimated* by the Global Chan-Vese method, due to a lower accuracy at locations characterised by strong, local greyscale variations. The image segmentation method based on the Local Chan-Vese energy functional generally curtails such inaccuracies, since it accounts for local image information that allows to adequately segment images with significant intensity inhomogeneity. Accordingly, the accuracy performance of the Local Chan-Vese method is superior to the Global Chan-Vese method and Otsu’s grey level thresholding method that only use global image information.

### Detailed features of cell morphology

**Fig. 9:**
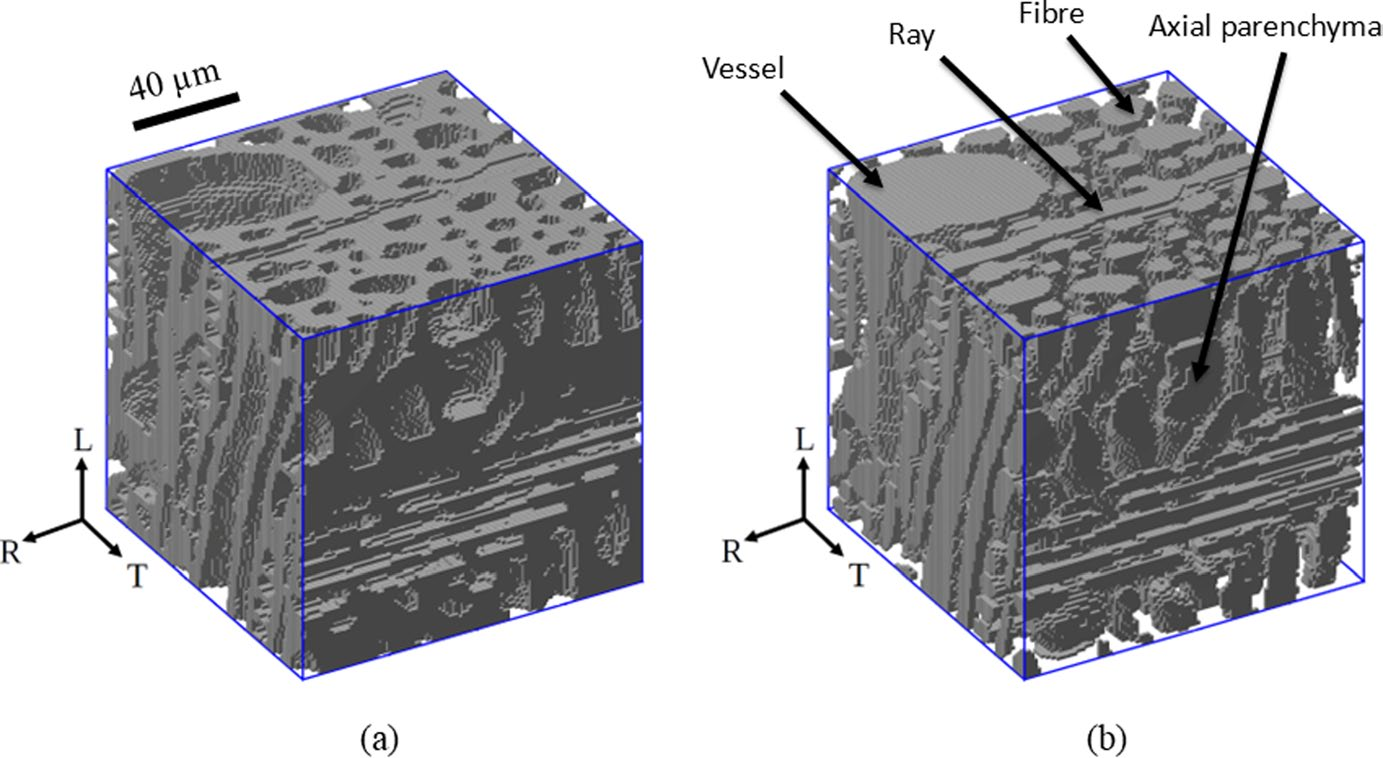
Image segmentation result of a *local latewood region* within an oak wood growth ring. The depicted micro-structural volume corresponds to the sample resulting from the convergence study in Fig. 6. **a** Binary image of the cell wall material (in grey), with the cell cavities occupying the space in between. **b** Binary image of the cell cavities (in grey), with the cell wall material occupying the intermediate space. The different cell types in the longitudinal direction (vessels, fibers, axial parenchyma) and radial direction (rays) can be clearly identified (colour figure online)

Figure 9 illustrates the segmented micro-structure of a local latewood region within an oak wood growth ring, as resulting from the convergence study displayed in Fig. 6, where Fig. 9a depicts the cell wall material in grey, with the cell cavities occupying the space in between, while Fig. 9b displays the cell cavities in grey, with the cell wall material occupying the intermediate space. The different cell types in longitudinal direction (fibers, axial parenchyma, vessels) and radial direction (rays) are also designated in Fig. 9b. In both figures the sharp, discernible phase boundaries and the high resolution of the depicted phases confirm the robustness and accuracy of the image segmentation method. In Fig. 9b the connections between the vertical, axial cells and the horizontal ray parenchyma cells can be also identified, which contribute to a *cell-cell communication network* that (i) efficiently stores and transports the water and mineral nutrients in wood, (ii) transfers signals about the physiological state of wood, producing responses such as the release of nutrients or stimulation of defense reactions in parenchyma, and (iii) facilitates the movement of growth regulators and molecules controlling gene expression within the cambium (i.e., a tissue layer that provides partially undifferentiated cells for growth in diameter) and within the zone of differentiating wood cells (Barnett 2006).

**Fig. 10:**
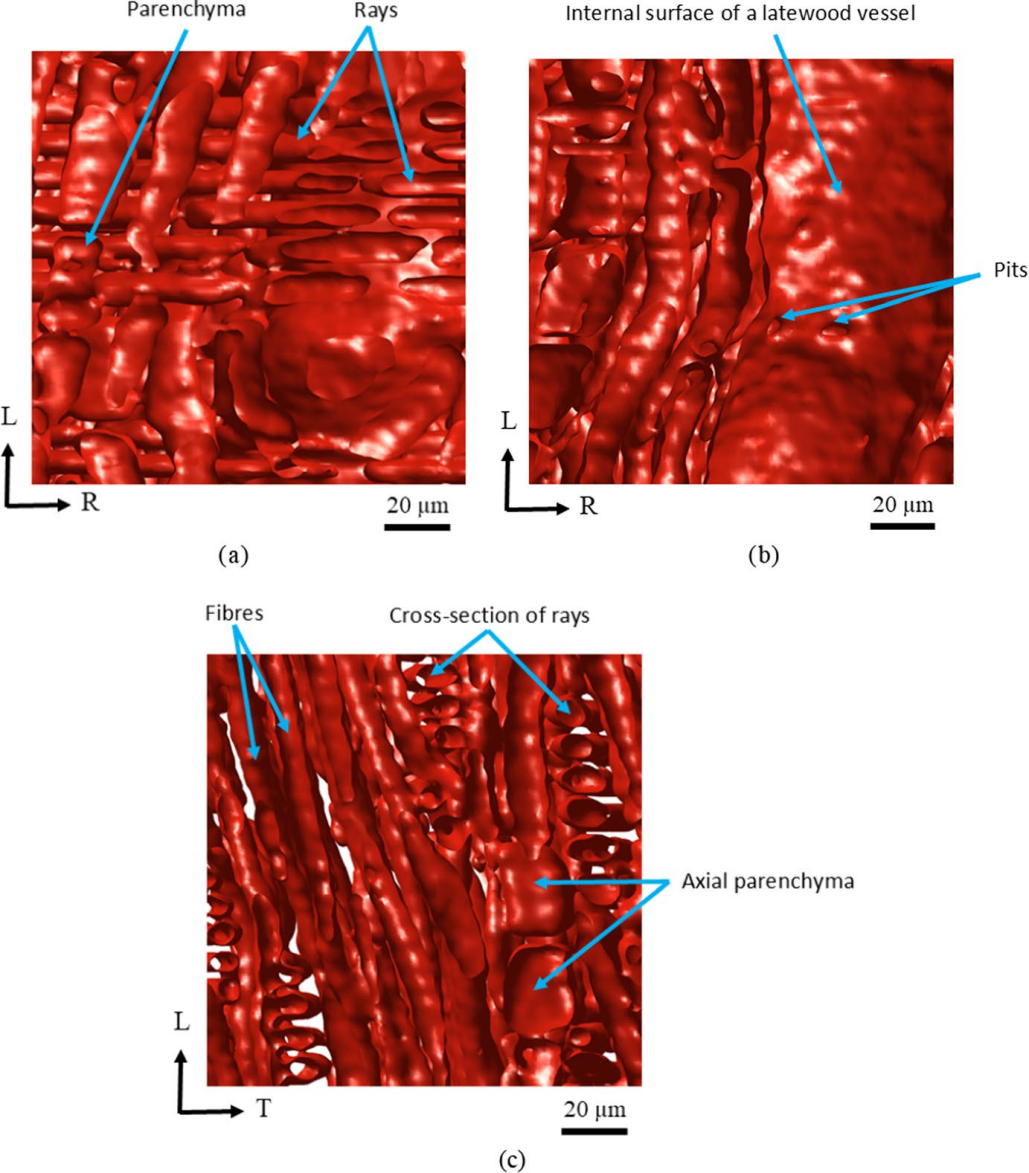
**a** Three-dimensional depth perspective of the cellular structure in the R-L plane of a *local latewood region* within an oak wood growth ring, showing individual rays and axial parenchyma. **b** 3D depth perspective of the cellular structure in the R-L plane, showing individual vessel with pits. **c** 3D depth perspective of cellular structure in the T-L plane, showing individual fibers, axial parenchyma and cross-sections of rays (colour figure online)

Figure 10 shows 3D depth perspectives of detailed morphological features of the cellular structure in the radial (R-L) and tangential (T-L) planes in a local latewood region of an oak growth ring. The perspective in the R-L plane depicted in Fig. 10a illustrates the multi-seriate rays oriented along the radial direction, and their connections with the axial parenchyma cells. The perspective in the R-L plane displayed in Fig. 10b shows a part of a vessel, which is mainly oriented in the longitudinal direction, and contains pits in the cell wall that serve as passages of communication with neighbouring cells. Pits come in pairs, i.e., one in each of the adjoining cell walls, separated by a membrane, and allow the flow of water and nutrients from one cell to another. In the T-L plane displayed in Fig. 10c individual fibres can be identified, which are predominantly oriented in the longitudinal direction and serve to provide mechanical resistance to the oak wood. Fibers typically have a smaller diameter and a larger length than the vessels responsible for water conduction. Furthermore, axial parenchyma, which consist of axially elongated cells, can be recognized, as well as the cross-sections of individual rays. In summary, the micro-structures presented in Fig. 10 clearly demonstrate that the level set-based image segmentation method is able to provide small-scale morphological features of oak wood cells in great detail.

### Comparison of computational cost on CPU and GPU hardware

In order to objectively compare the computational cost of the image segmentation method presented in Sect. "Level set-based image segmentation method" on CPU and GPU hardware, the method has been implemented in the open-source Python programming environment. The standard and optimized functions and types within the PyTorch library of Python allow to create a robust interface for a seamless execution on both CPU and GPU architectures with minimal modifications of the programming script. For an efficient computation, the CPU implementation benefits from shared memory parallelism using backend libraries (i.e., OpenMP), while the GPU implementation benefits from the parallel architecture of GPUs and the CUDA library. The computations were performed using the CPU hardware “AMD EPYC 7452 32-Core Processor”, which consists of 4 NUMA regions on a single socket that include a total of 32 cores with shared memory access. Further, an NVIDIA A40 GPU was selected for its powerful parallel processing capabilities, enabling the efficient computation of dense matrix operations.

**Fig. 11:**
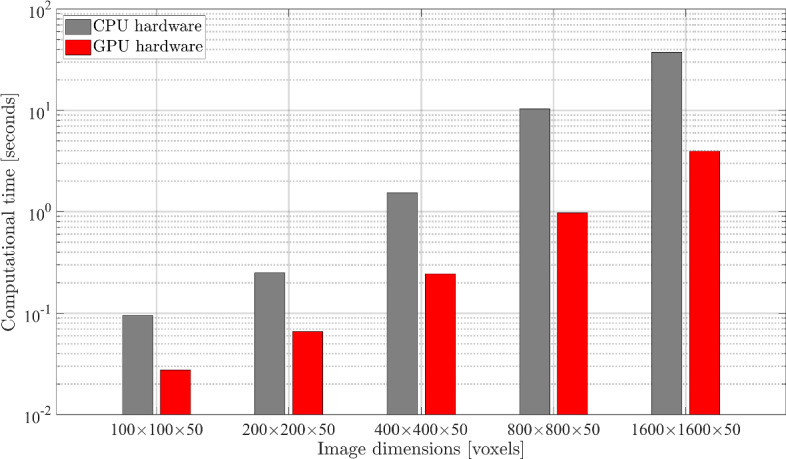
Comparison of the computational cost of the image method segmentation method on CPU (grey bar) and GPU (red bar) hardware. Five different images are considered, with sizes of $100\times 100\times 50$ voxels, $200\times 200\times 50$ voxels, $400\times 400\times 50$ voxels, $800\times 800\times 50$ voxels, and $1600\times 1600\times 50$ voxels (colour figure online)

The comparison of the computational cost of the image segmentation method on CPU and GPU hardware is presented in Fig. 11 considering images of 5 different sizes, i.e., $100\times 100\times 50$ voxels, $200\times 200\times 50$ voxels, $400\times 400\times 50$ voxels, $800\times 800\times 50$ voxels, and $1600\times 1600\times 50$ voxels. The number of increments used in the image segmentation process is set equal to 10; based on the result in Fig. 6, this somewhat conservative value is expected to lead to an accurate segmentation result for each of the 5 image sizes. Note that the computational time of the segmentation process of a specific image may vary somewhat if the process is executed multiple times. For CPU hardware this is due to a variability in memory allocation, the effect of background processes, and fluctuations in computational overhead. For GPU hardware these effects may also influence the computational time, but typically to a (far) lesser extent, as GPU kernels can execute computations asynchronously. To account for the variability in computational time, for each image size the segmentation process was performed 20 times, and the average computational time is displayed in Fig. 11. Although the CPU hardware already leads to adequate computation times, the computational cost on the GPU hardware is noticeably lower, as shown by the relative reduction in computational time by factors of 3, 4, 6, 10, and 10 under the stepwise increase in image size. Compared with the CPU architecture, the GPU architecture more effectively divides the computational task into (many) small components that are executed in parallel with high arithmetic intensity, resulting in a growing difference in computational efficiency under increasing image size.

### Statistical information on inner cell diameters and cell wall thickness

**Fig. 12:**
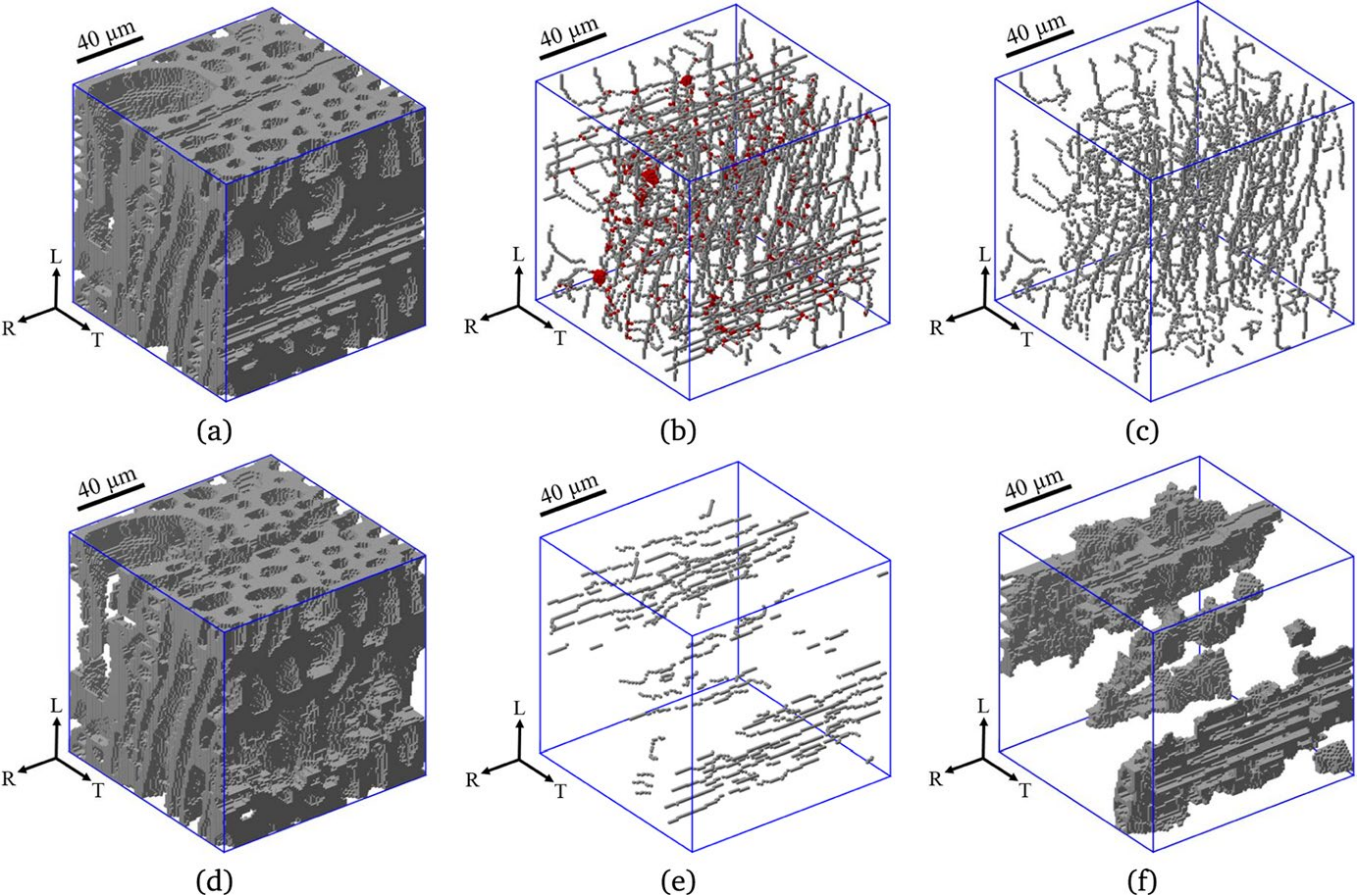
Skeleton-based image segmentation procedure of a *local latewood region* into axial cells and radial cells. **a** Binary image of cell wall material (in grey), with the cell cavities occupying the space in between. **b** Skeleton of both axial cells and radial cells. The interconnections between axial cells and radial cells are indicated by the red voxels. **c** Skeleton of only axial cells. **d** Cell wall material of only axial cells (in grey). **e** Skeleton of only radial cells. **f** Cell wall material of only radial cells (in grey) (colour figure online)

A statistical analysis is carried out of the maximum and minimum inner cell diameters $d_{max}$ and $d_{min}$ and the cell wall thickness *t* of the various cell types present in the axial (longitudinal) direction and radial direction of micro-structural samples A and B. The distinct axial and radial cells following from the skeleton-based image segmentation step described in Sect. "Skeleton-based image segmentation into axial cells and ray parenchyma cells" are illustrated in Fig. 12 for a local latewood region in one of the two oak wood samples. The geometrical properties of the axial cells (fibers, axial parenchyma, earlywood vessels, latewood vessels) and radial cells (ray parenchyma) were measured from a number of cross-sections in the R-T plane and in the T-L plane, respectively. In specific, in the axial direction 10 cross-sectional images were taken at a mutual distance of 6.5 $\mu$m, which cover the complete sample thickness of $\mathcal {T}$ = 65 $\mu$m listed in Table 1, while in the radial direction 30 cross-sectional images were taken at a mutual distance of 65 $\mu$m, which cover 75% of the sample width $\mathcal {W}$ = 2600 $\mu$m. The statistical results for the maximum and minimum inner cell diameters of the different cell types are presented in Sect. "Maximum and minimum inner cell diameters", and the statistical results for the cell wall thickness can be found in Sect. "Cell wall thickness".

#### Maximum and minimum inner cell diameters

**Fig. 13:**
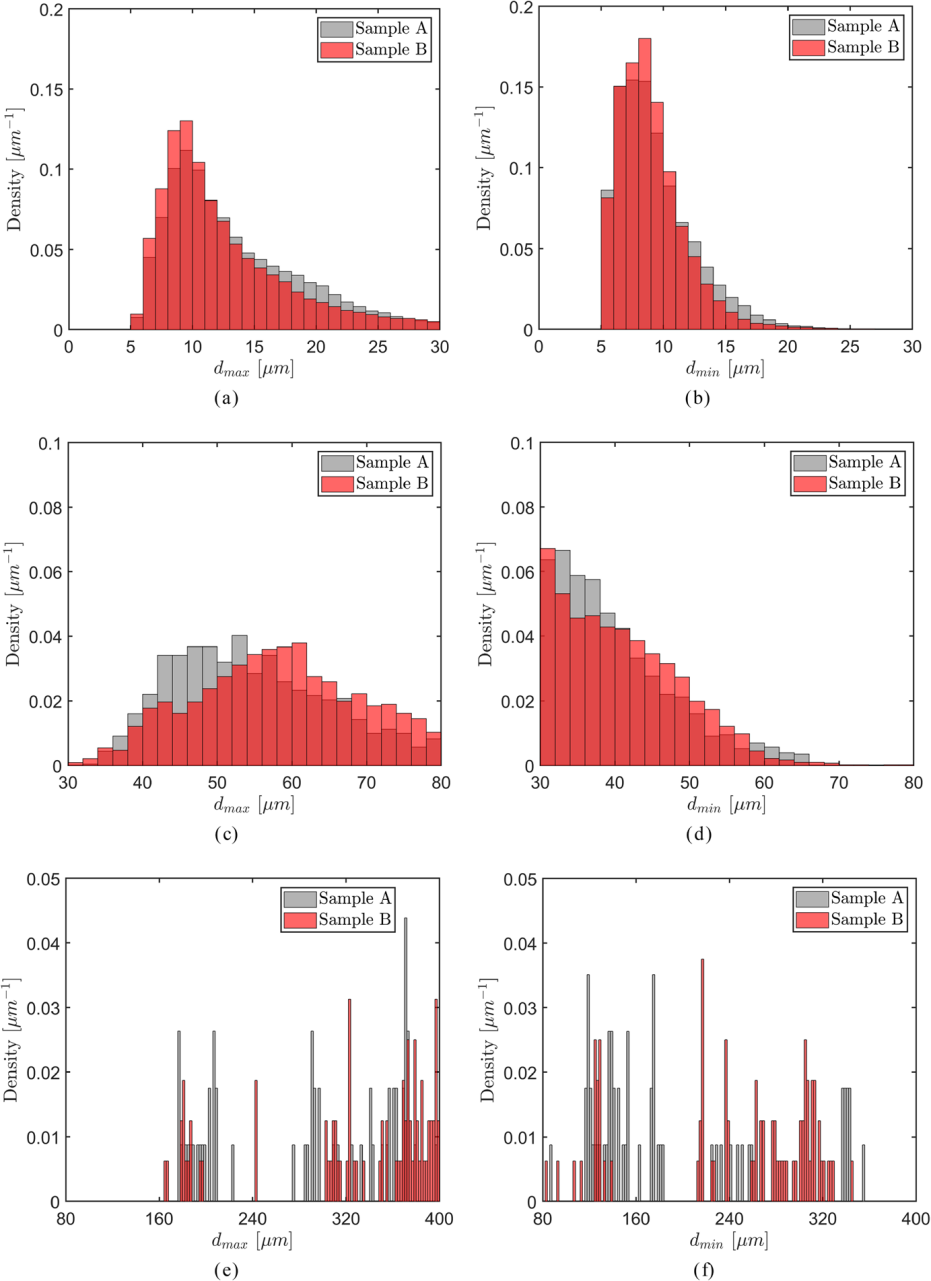
Density histograms of the maximum inner diameter $d_{max}$ (left column of figures) and the minimum inner diameter $d_{min}$ (right column of figures) of the *axial cells* in the oak samples A (grey bars) and B (red bars). **a** $d_{max}$ for *small* cell diameters. **b** $d_{min}$ for *small* cell diameters. **c** $d_{max}$ for *medium* cell diameters. **d** $d_{min}$ for *medium* cell diameters. **e** $d_{max}$ for *large* cell diameters. **f** $d_{min}$ for *large* cell diameters (colour figure online)

The axial cells oriented in the *longitudinal direction* are characterized by a wide range of diameters, $[ 0 - 400 \mu \text{m})$, where the larger cells with diameters above $30 \mu \text{m}$ appear at *significantly lower* frequencies than the relatively small cells with diameters less than $30 \mu \text{m}$, see also Fig. 5. Consequently, a density histogram that includes the whole range of cell diameters $[ 0 - 400 \mu \text{m})$ will only adequately show statistical information regarding cell diameters smaller than $30 \mu \text{m}$, and therefore is of limited use. For this reason, for the axial cells the density histograms of the maximum and minimum inner cell diameters, $d_{max}$ and $d_{min}$, are shown in Fig. 13 for three, *separate* consecutive ranges, namely *small* cell diameters - $[ 0 - 30 \mu \text{m})$, *medium* cell diameters - $[ 30 - 80 \mu \text{m})$, and *large* cell diameters - $[ 80 - 400 \mu \text{m})$, with the left column of figures—Fig. 13a, c and e—showing the maximum inner cell diameter $d_{max}$ for the three ranges, and the right column of figures—Figs. 13(b), (d) and (f)—showing the minimum inner cell diameter $d_{min}$. The grey and red coloured bars used in the density histograms respectively refer to samples A and B, of which the properties are listed in Table 1; the light red colour used for part of the bars denotes how much larger the corresponding density of sample B is with respect to that of sample A. The minimum bar width applied equals 1 $\mu$m, which approximately corresponds to the voxel width of 1.3 $\mu$m used for creating the images of the oak micro-structures. In accordance with the common convention, the area under the density histograms equals unity.

The diameter range $[ 0 - 30 \mu \text{m})$ depicted in Fig. 13a and b refers to axial cells with relative small inner diameters, i.e., *fibers* and *axial parenchyma*. Observe that the density histograms obtained from samples A and B are comparable; the maximum and minimum inner cell diameters start at a value of 5 $\mu$m, and are characterized by a most frequent value between 7 and 10 $\mu$m. These values are in good agreement with the average inner diameters of 10 and 11 $\mu$m reported in Maeglin and Quirk (1984) for, respectively, 3 commercial red oaks and 8 commercial white oaks, as measured from microscopy images of 41 specimens. The average inner diameters reported in this reference for the axial parenchyma are only slightly larger, and equal 14 $\mu$m and 15 $\mu$m for the white oak and red oak samples, respectively. The fact that the density histogram of $d_{max}$ continues until its final value of 30 $\mu$m, while the density histogram of $d_{min}$ more or less terminates after reaching a value of 22 $\mu$m, indicates that (a part of) the fibers and parenchyma have an oval-shape cross-section. Note that the range of inner diameters of the density histograms displayed in Fig. 13a and b includes the dimensions of the fibers and axial parenchyma appearing in the latewood region depicted in Fig. 9b.

The diameter range $[ 30 - 80 \mu \text{m})$ considered in Fig. 13c and d refers for a large extent to *latewood vessels*. This observation is in agreement with Chavenetidou et al. (2024), where for latewood vessels the average diameter was reported to fall in the range of 35–84 $\mu$m, based on microscopy analyses of four different oak species. The diameters of the latewood vessels in samples A and B show similar distributions, with the most frequent value of $d_{max}$ falling in the range of 52–56 $\mu$m, and the most frequent value of $d_{min}$ being equal to 30 $\mu$m. Note that the above ranges of values reflect the dimensions of the latewood vessel depicted in Fig. 9b.

The density histograms in Fig. 13e and f representative of the cell diameter range $[ 80 - 400 \mu \text{m})$ show a discontinuous distribution of both $d_{max}$ and $d_{min}$, as characterized by relatively large intervals in which no data appears. The density histograms of $d_{max}$ start at a value between 160 $\mu$m and 180 $\mu$m, and for samples A and B are clearly constructed by different diameter values. These two features are characteristic of *earlywood vessels*, where the intervals with no data originate from the relatively low number of vessels present in the micro-structural samples. The absence of data for $d_{max}$ at the beginning of the domain and the absence of data for $d_{min}$ at the end of the domain (especially for sample B) indicate that (part of) the earlywood vessels have a (slightly) oval-shaped cross-section. It is further noted that the diameter distributions depicted in Fig. 13e and f include the range of average diameters of 201 - 332 $\mu$m reported in Chavenetidou et al. (2024) for earlywood vessels in oak.

**Fig. 14:**
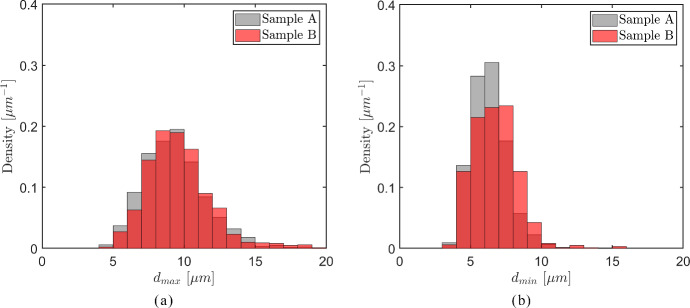
Density histograms of the maximum and minimum inner diameters of the *radial cells* in the oak samples A (grey bars) and B (red bars). **a** Maximum cell diameter $d_{max}$. **b** Minimum cell diameter $d_{min}$ (colour figure online)

For the cells oriented in the *radial direction*, the density histograms of the inner diameters $d_{max}$ and $d_{min}$ are depicted in Fig. 14a and b, considering a range $[ 0 - 20 \mu \text{m})$ for both diameters. It shows that the density histograms for $d_{max}$ are comparable for samples A and B, while the density histograms for $d_{min}$ somewhat differ in the central part. Observe that the most frequent value of $d_{max}$ lies between 8 and 10 $\mu$m, and of $d_{min}$ lies between 6 and 8 $\mu$m. The radial cells mostly refer to *ray parenchyma*, of which cross-sectional areas with similar dimensions can be seen in Fig. 10c. Since the relative shift between the density histograms of $d_{max}$ en $d_{min}$ is small, it may be concluded that the cross-sectional shape of the rays is close to circular. The average inner diameters reported in Maeglin and Quirk (1984) for ray parenchyma in red and white oak lie in between 11 and 13 $\mu$m, and thus lie slightly above the above-mentioned ranges of most frequent values for $d_{max}$ and $d_{min}$.

#### Cell wall thickness

**Fig. 15:**
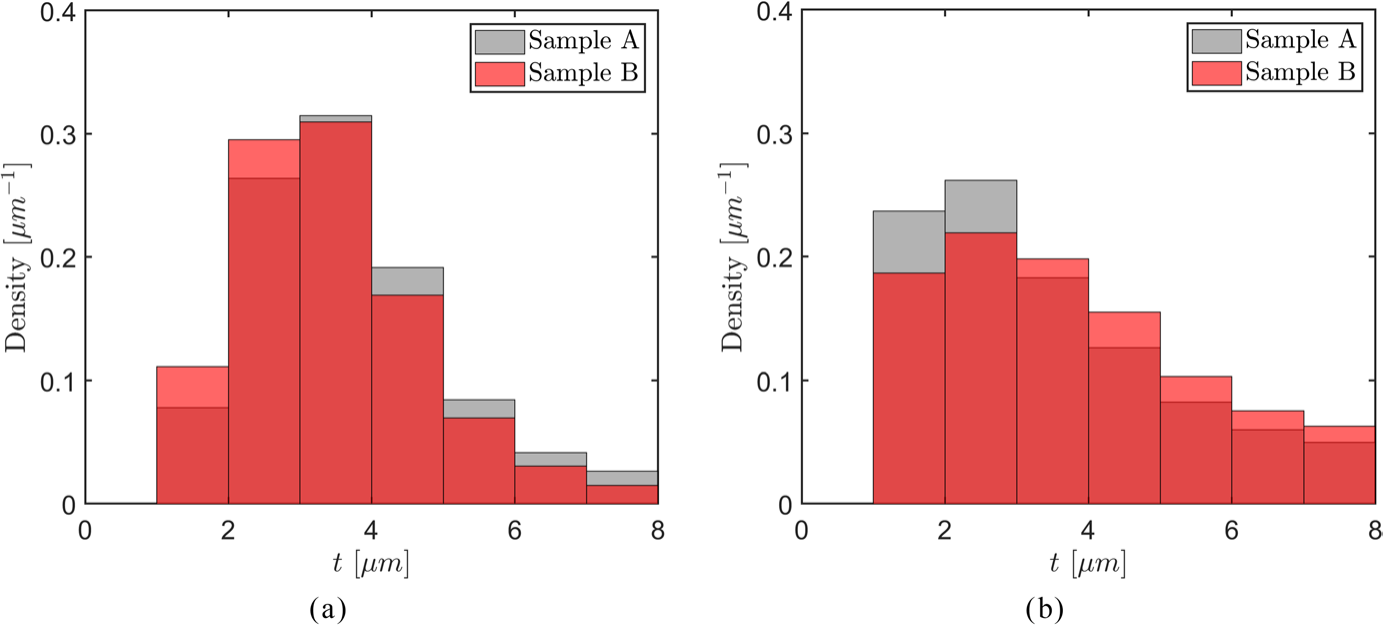
Density histograms of the cell wall thickness *t* of the *axial cells* and *radial cells* in the oak samples A (grey bars) and B (red bars). **a** Axial cells. **b** Radial cells (colour figure online)

The density histograms of the cell wall thickness *t* of the axial and radial cells are displayed in Fig. 15a and b, respectively. Recall that the cell wall thickness refers to the sum of the thicknesses of the three secondary cell wall layers and half of thickness of the compound middle lamella layer, see also Fig. 4e. The density histograms of the cell wall thicknesses of samples A and B are comparable, ranging from 1 to 8 $\mu$m, with the most frequent value appearing between 2 and 4 $\mu$m. This range closely resembles the range of average cell wall thicknesses of 2.2–3.5 $\mu$m reported in Maeglin and Quirk (1984) for the combination of fibers, axial parenchyma, earlywood vessels and ray parenchyma in white oak samples. For the red oak samples analyzed in this reference, the range of average cell wall thicknesses for the combination of fibers, axial parenchyma, and ray parenchyma is comparable, while the earlywood vessels have a somewhat larger average cell wall thickness of 6 $\mu$m. The above range of most frequent values further resembles the range of average cell wall thicknesses of 1.4–3.9 $\mu$m reported in Kim and Daniel (2016), which was obtained from microscopy images by computing the sum of the thicknesses of the three secondary cell wall layers of cells in both the earlywood and latewood regions of oak growth rings. Note that this measure for the cell wall thickness does not incorporate half of the thickness of the compound middle lamella layer accounted for in the thickness *t*; however, for both the axial cells and ray cells in oak wood the relative contribution of the compound middle lamella layer to the total wall thickness of cells is only about 5% (Livani et al. 2023).

Tables 2 and 3 list the average value $\mu _s$ and standard deviation $\sigma _s$ of the inner diameters $d_{max}$ and $d_{min}$ and the cell wall thickness *t* of the different cell structures considered in Figs. 13–15. When comparing the statistical values for samples A and B, both the average diameters and the standard deviations of the radial cells and small-size and medium-size axial cells are close in magnitude, which is in agreement with the resemblance of their density histograms, see Figs. 13a-d and 15. Conversely, for the large-size axial cells the average cell diameters of samples A and B somewhat differ, which may be ascribed to the large variability in cell diameter and the relatively low frequency of appearance of earlywood vessels in a single growth ring, see Figs. 13e and f and Fig. 5. Observe further that the average cell wall thickness and standard deviation of the axial and radial cells of Samples A and B are also comparable. In summary, it may thus be concluded that the micro-structural geometries of Samples A and B are similar in terms of most of the descriptive statistical values listed in Tables 2 and 3, with some difference regarding the average cell diameters of large-size axial cells.

**Table 2:** Average value $\mu _s$ and standard deviation $\sigma _s$ of the inner cell diameters $d_{max}$ and $d_{min}$ of the various cell structures characterizing samples A and B, see Figs. 13 (axial cells) and 14 (radial cells) for the corresponding density histograms

		Sample A		Sample B	
		$\mu _s$ [$\mu$m]	$\sigma _s$ [$\mu$m]	$\mu _s$ [$\mu$m]	$\sigma _s$ [$\mu$m]
*Axial cells*					
Small cell diameter	$d_{max}$	13.2	5.2	12.4	5.0
$\, \, \, [0 - 30 \mu \text{m})$	$d_{min}$	9.4	3.1	9.0	2.6
Medium cell diameter	$d_{max}$	54.4	10.4	57.9	10.8
$\, \, \, [30 - 80 \mu \text{m})$	$d_{min}$	40.0	7.8	40.9	8.0
Large cell diameter	$d_{max}$	289.1	75.5	327.1	73.7
$\, \, \, [80 - 400 \mu \text{m})$	$d_{min}$	188.5	77.7	244.0	72.3
*Radial cells*					
Cell diameter	$d_{max}$	9.3	2.2	9.6	2.3
$\, \, \, [0 - 20 \mu \text{m})$	$d_{min}$	6.3	1.3	6.7	1.6

**Table 3:** Average value $\mu _s$ and standard deviation $\sigma _s$ of the cell wall thickness *t* of the axial cells and radial cells characterizing samples A and B, see Fig. 15 for the corresponding density histograms

		Sample A		Sample B	
		$\mu _s$ [$\mu$m]	$\sigma _s$ [$\mu$m]	$\mu _s$ [$\mu$m]	$\sigma _s$ [$\mu$m]
*Axial cells*					
Cell wall thickness $[ 0 - 8 \mu \text{m})$	*t*	3.6	1.4	3.4	1.3
*Radial cells*					
Cell wall thickness $[ 0 - 8 \mu \text{m})$	*t*	3.4	1.7	3.7	1.8

The statistical analysis of the inner cell diameters and cell wall thickness of the various cell types in oak wood provides an efficient and accurate characterization of the oak micro-structure, and the results obtained generally are in good correspondence with other experimental data reported in the literature. The density histograms constructed appear to be useful for a straightforward comparison of the morphological features of different oak samples, which may help to better understand the variability in their mechanical/physical properties and adequately classify various oak species. It is emphasized, however, that the present statistical analysis is not exhaustive, and mainly serves to demonstrate the accuracy and practical applicability of the results obtained with the current image segmentation method. In fact, for a further characterization of the oak micro-structure the statistical analysis can be extended with the deduction of additional micro-structural descriptors, such as *n*-point probability functions, surface correlation functions, lineal-path functions, chord-length density functions, as well as with detailed analyses of the statistical inhomogeneity, statistical anisotropy and ergodicity of the micro-structural samples (Torquato 2002). Such analyses fall beyond the scope of the present study, and would require the investigation of more than two micro-structural samples in order to rigorously ensure that all results obtained are statistically representative.

## Conclusion

A level set-based image segmentation method is presented that can be used for a robust identification and accurate characterization of the different cell types defining complex wood micro-structures. The method can be applied to arbitrary wood species, and in this contribution is elaborated for oak. The evolution of the level set function $\phi$ used in the image segmentation method is described by an Euler-Lagrange equation, which follows from defining an appropriate “energy” functional and minimizing this functional with respect to $\phi$ within a variational framework. The variational framework is based on the Local Chan-Vese energy functional, which is an extension of the original Chan-Vese energy functional that incorporates, in addition to *global* image information, *local* image information to adequately segment images with significant intensity inhomogeneity. The discretized evolution equation and boundary conditions derived from the variational framework contain specific features that are different, or absent, in the original model formulations. The application of the level-set image segmentation approach enables to accurately distinguish cell wall material from cell cavities. The cell material objects are subsequently segmented into axial cell objects and ray parenchyma cell objects oriented in the longitudinal and radial material directions of oak wood, respectively. This is done by converting the corresponding cell cavity objects to their medial axes of a single voxel wide to obtain their skeleton, after which the cell wall material of the axial cells with a skeleton (mainly) oriented in the longitudinal material direction can be straightforwardly separated from that of the ray cells with a skeleton (mainly) oriented in the radial material direction. This additional segmentation step facilitates the collection of statistical information on the inner cell dimensions and wall thickness of axial cells and ray parenchyma cells from images taken across the principal material planes of the oak micro-structure.

The performance and results of the above image segmentation method are analyzed by using as input detailed micro-structural images of two representative oak samples containing a single growth ring, as obtained from X-ray micro-computed tomography experiments. The assessment of the robustness and convergence behaviour of the image segmentation method shows that the method converges very fast—within a few incremental steps—into a unique oak micro-structure that is independent of the chosen initial configuration. The high accuracy of the image segmentation method is demonstrated by visualizing small-scale morphological features of oak micro-structures in great detail. The relative accuracy of the method is assessed through a comparison of the results with those obtained by two other image segmentation methods presented in the literature, which are Otsu’s grey level thresholding method, and the Global Chan-Vese method. The accuracy performance of the Local Chan-Vese method turns out to be superior to both the Global Chan-Vese method and Otsu’s grey level thresholding method. This is, because the latter two methods only consider global image information, while the Local Chan-Vese method also takes into account local image information, making it possible to accurately segment images with significant intensity inhomogeneity. The computational cost of the image segmentation method is evaluated by comparing its performance on CPU and GPU hardware. Although the CPU hardware already leads to adequate computational times, the computational cost on the GPU hardware is noticeably lower, where the difference in computational efficiency grows with increasing image size (up to a factor of 10 for the images considered in the comparison study). Additionally, a statistical analysis is carried out to demonstrate the practical applicability of the results obtained by the current image segmentation method. The maximum and minimum inner cell diameters and the cell wall thickness are determined for the various axial cells—fibers and axial parenchyma, earlywood vessels, latewood vessels—and ray parenchyma cells defining the micro-structure of the oak growth ring samples. The density histograms constructed for these geometrical parameters provide their statistical spread and most frequent value, which are quite similar for the two oak samples and are in good agreement with other experimental data reported in the literature.

The presented image segmentation method and results are of specific interest for the wood science community, enabling the analysis of the mechanical and physical properties of oak wood based on detailed input of the actual micro-structure. Moreover, the developed method provides new opportunities for a detailed micro-structural study of other wood species.
